# Mapping the evidence on pharmacological interventions for non-affective psychosis in humanitarian non-specialised settings: a UNHCR clinical guidance

**DOI:** 10.1186/s12916-017-0960-z

**Published:** 2017-12-11

**Authors:** Giovanni Ostuzzi, Corrado Barbui, Charlotte Hanlon, Sudipto Chatterjee, Julian Eaton, Lynne Jones, Derrick Silove, Peter Ventevogel

**Affiliations:** 1 WHO Collaborating Centre for Research and Training in Mental Health and Service Evaluation, Department of Neuroscience, Biomedicine and Movement Sciences, Section of Psychiatry, University of Verona, Piazzale L.A. Scuro 10, 37134 Verona, Italy; 2 Addis Ababa University, College of Health Sciences, School of Medicine, Department of Psychiatry, 6th Floor College of Health Sciences Building, Tikur Anbessa Hospital, PO 9086, Addis Ababa, Ethiopia; 3 King’s College London, Institute of Psychiatry, Psychology and Neuroscience, Centre for Global Mental Health, London, UK; 4 Sangath Centre, Porvorim, Goa India; 5 School of Population Health, Melbourne, Australia; 6 London School of Hygiene and Tropical Medicine, Keppel Street, London, UK; 7 CBM International, Bensheim, Germany; 8 FXB Center for Health & Human Rights, Harvard University, Boston, USA; 9 Psychiatry Research and Teaching Unit, School of Psychiatry, University of New South Wales, Sydney, Australia; 10 Public Health Section, United Nations High Commissioner for Refugees, Geneva, Switzerland

**Keywords:** Global mental health, Humanitarian settings, Antipsychotics, Non-affective psychosis, Translational research, Clinical guidance

## Abstract

**Background:**

Populations exposed to humanitarian emergencies are particularly vulnerable to mental health problems, including new onset, relapse and deterioration of psychotic disorders. Inadequate care for this group may lead to human rights abuses and even premature death. The WHO Mental Health Gap Action Programme Intervention Guide (mhGAP-IG), and its adaptation for humanitarian settings (mhGAP-HIG), provides guidance for management of mental health conditions by non-specialised healthcare professionals. However, the pharmacological treatment of people with non-affective psychosis who do not improve with mhGAP first-line antipsychotic treatments is not addressed. In order to fill this gap, UNHCR has formulated specific guidance on the second-line pharmacological treatment of non-affective psychosis in humanitarian, non-specialised settings.

**Methods:**

Following the Grading of Recommendations, Assessment, Development and Evaluation (GRADE) methodology, a group of international experts performed an extensive search and retrieval of evidence on the basis of four scoping questions. Available data were critically appraised and summarised. Clinical guidance was produced by integrating this evidence base with context-related feasibility issues, preferences, values and resource-use considerations.

**Results:**

When first-line treatments recommended by mhGAP (namely haloperidol and chlorpromazine) are not effective, no other first-generation antipsychotics are likely to provide clinically meaningful improvements. Risperidone or olanzapine may represent beneficial second-line options. However, if these second-line medications do not produce clinically significant beneficial effects, there are two possibilities. First, to switch to the alternative (olanzapine to risperidone or vice versa) or, second, to consider clozapine, provided that specialist supervision and regular laboratory monitoring are available in the long term. If clinically relevant depressive, cognitive or negative symptoms occur, the use of a selective serotonin reuptake inhibitor may be considered in addition or as an alternative to standard psychological interventions.

**Conclusions:**

Adapting scientific evidence into practical guidance for non-specialised health workers in humanitarian settings was challenging due to the paucity of relevant evidence as well as the imprecision and inconsistency of results between studies. Pragmatic outcome evaluation studies from low-resource contexts are urgently needed. Nonetheless, the UNHCR clinical guidance is based on best available evidence and can help to address the compelling issue of undertreated, non-affective psychosis in humanitarian settings.

**Electronic supplementary material:**

The online version of this article (doi:10.1186/s12916-017-0960-z) contains supplementary material, which is available to authorized users.

## Background

There has been a dramatic increase in worldwide humanitarian emergency situations in recent years, provoked by forced displacement related to armed conflicts and persecution as well as to environmental disasters, including drought, flooding and earthquakes. People in such humanitarian settings have elevated risks for the development of mental health issues, which cause additional suffering and constitute major clinical and public health concerns [1–6]. In humanitarian emergencies, mental health issues are at risk of being overlooked [7]. In particular, while many efforts have been made to describe and address stress-related disorders, such as post-traumatic stress disorder, anxiety and emotional disorders, including depression, far less attention has been given to the epidemiological characterisation and clinical management of non-affective psychosis (including schizophrenia) [8–15]. Thus, there is a major treatment gap, particularly considering that epidemiological data suggest that the prevalence of psychotic disorders is heightened in refugees in comparison with both native populations and non-refugee migrants [12], and that, in humanitarian emergencies, people with pre-existing psychosis are particularly vulnerable to relapse and deterioration [16–18]. In humanitarian settings, people with psychotic disorders constitute a significant proportion of the caseload in clinical mental health programmes, with rates ranging from 8.6% to 41.2% of overall mental disorders [19–23]. These individuals are particularly vulnerable to human rights violations, discrimination, social exclusion and even premature death [24–26].

In the last 10 years, efforts have been made to provide non-specialised healthcare professionals with easily accessible tools for managing mental health conditions of high priority. The World Health Organization (WHO) and the United Nations High Commissioner for Refugees (UNHCR) have developed policies and tools to expand access to mental healthcare to underserved populations through the decentralisation of basic mental healthcare and integration of mental health into primary care [27, 28]. In particular, the Mental Health Gap Action Programme Intervention Guide (mhGAP-IG) [29, 30] and the Mental Health Gap Action Programme Humanitarian Intervention Guide (mhGAP-HIG) [31] represent successful examples of this approach. However, neither mhGAP-IG nor mhGAP-HIG address the management of people with long-term, disabling mental disorders, particularly non-affective psychosis, who remain symptomatic after antipsychotic treatment provided according to mhGAP guidelines. Although the number of people with treatment-resistant psychosis may be relatively small, the unmet mental health needs of this group lead to significant social and economic burden for families, health workers, and the wider community. Furthermore, for this population, guidance on subsequent pharmacological options is limited.

In order to fill this gap, UNHCR has recently formulated specific guidance on the pharmacological treatment of non-affective psychosis in humanitarian non-specialised settings. This paper anticipates the methodology employed to evaluate and summarise the best available evidence, and reports on how the evidence was translated into pragmatic guidance for healthcare professionals. The expected impact of the UNHCR guidance in humanitarian settings, as well as the potential obstacles to its effective implementation, are also discussed.

## Methods

The Grading of Recommendations, Assessment, Development and Evaluation (GRADE) methodology [32] guided the process from evidence retrieval to the production of a pragmatic guidance for health professionals working in humanitarian settings. A scientific secretariat, represented by the WHO Collaborating Centre for Research and Training in Mental Health and Service Evaluation in Verona, Italy, worked closely with an advisory panel of international experts with in-depth expertise spanning the fields of clinical psychopharmacology, mental health systems and services research in humanitarian settings, health policy development, health economics, and implementation science. According to the GRADE methodology, key scoping questions for this guidance were formulated on the basis of a shared process directly involving in-field experts and practitioners, with a strong emphasis on the role of specific context variables [33]. These scoping questions guided evidence retrieval, critical appraisal and interpretation:

1. In people with non-affective psychosis who do not improve after treatment with a first-generation antipsychotic (FGA), is switching to another FGA effective and safe?
2. Are second-generation antipsychotics (SGAs) effective and safe in people with non-affective psychosis who do not improve with FGAs used as first-line treatment?
3. Which antipsychotic is effective and safe in people with a diagnosis of treatment-resistant non-affective psychosis?
4. Are antidepressant-antipsychotic combinations effective and safe in people with non-affective psychosis who develop depressive, cognitive and negative symptoms?

**Fig. 1:**
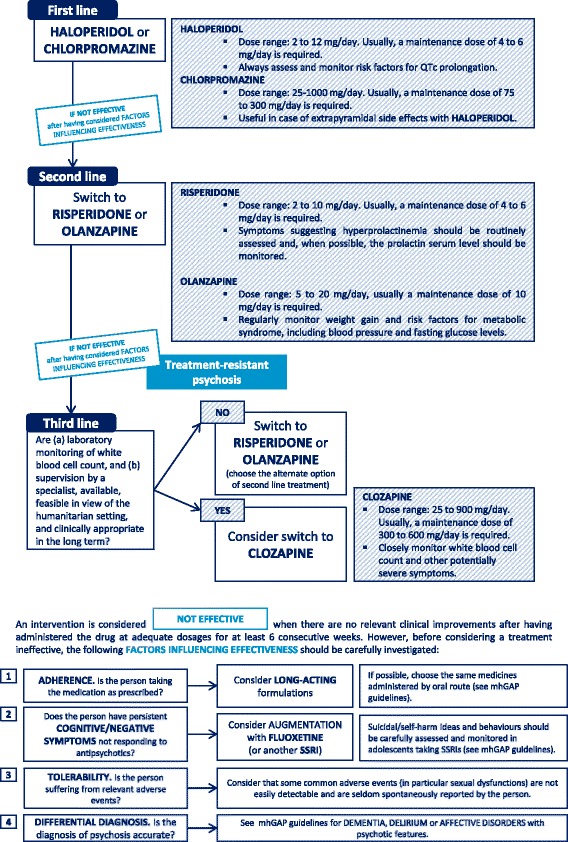
Flow-chart describing the clinical pathway for the choice of antipsychotics

In order to address each question, the target populations, settings, interventions and outcomes of interest were characterised by employing a Population, Intervention, Comparison, Outcomes framework. In order to extensively review all available data on the pharmacological treatments of non-affective psychosis, for each Population, Intervention, Comparison, Outcomes table we systematically searched electronic databases (PubMed, PsychINFO, CINHAL, MEDLINE, Web Of Science Core Collection, the Cochrane Central Register of Controlled Trials) to identify the most recent good-quality systematic review for each intervention of interest. We used the terms “psychosis OR psychotic OR schizophrenia” in association with (1) specific search filters for systematic reviews and meta-analyses (http://hiru.mcmaster.ca/hiru/HIRU_Hedges_MEDLINE_Strategies.aspx#Reviews); (2) the specific term identifying the subgroup of interest (e.g. “treatment-resistant”, “negative symptoms”); and (3) the names of the medications of interest. No language restrictions were applied. The last update of the search was performed in January 2017. We included only systematic reviews and/or meta-analysis of randomised controlled trials including adult patients. When more than one review provided data for the same outcome, the most recent and comprehensive review was chosen. When systematic reviews of randomised trials were not available, we searched for the most up-to-date and good quality individual randomised trials and observational studies. The scientific secretariat summarised the results of the included reviews and assessed their quality by employing the Guideline Development Tool [34], an online software that helps produce evidence summaries and healthcare recommendations according to the GRADE approach. The GRADE tables produced are available as Additional file 1. On the basis of evidence summaries, in line with the GRADE methodology, the panel critically discussed the balance between the possible clinical advantages and disadvantages of different treatment options, considering context-related feasibility issues, costs, ethical issues, values, preferences and insights from experts working in low-resource settings. This led to the development of the clinical practice guidance, graphically summarised in Fig. 1. Doses were reported according to licensed doses from the British National Formulary [35]. When the licensed dose range was considered to possibly diverge from that used in common clinical practice, we employed data from the most updated systematic reviews or guidelines. Further, additional clinical annotations (including the use of long-acting formulations and relevant insights on the monitoring and management of adverse events) were derived from the mhGAP and, if needed, from the most updated international guidelines and regulatory documents in order to pragmatically support mental health professionals in routine practice. These pragmatic annotations will be available in the final format of the UNHCR guidance. Additionally, a comprehensive evidence summary reporting each step of this process will be made available online for consultation.

## Results

### In people with non-affective psychosis who do not improve after treatment with a FGA, is switching to another FGA effective and safe?

Haloperidol and chlorpromazine were used as the reference standard, as these medications are the first-line recommended treatments for patients with non-affective psychosis in the mhGAP guidelines [29, 36].

We did not find studies of SGA intervention in individuals who had failed to improve after treatment with one FGA. Therefore, we included studies conducted in the general population of people suffering from non-affective psychosis. This evidence was rated as indirect (Additional file 1).

According to available evidence, no difference in treatment response was identified between haloperidol and FGAs as a class, and between haloperidol and the following medications considered individually: chlorpromazine, perphenazine, pimozide, fluphenazine and trifluoperazine. Haloperidol showed a slightly better overall acceptability with respect to chlorpromazine, but caused more movement disorders. Perphenazine and FGAs as a class did not show benefits compared with haloperidol in terms of efficacy, acceptability and tolerability. Pimozide, fluphenazine and trifluoperazine did not show benefits over haloperidol in terms of efficacy, while data on acceptability and tolerability were not available.

With few exceptions, the GRADE tables showed ‘low’ or ‘very low’ quality for the vast majority of outcomes, mostly due to the indirectness of the evidence, as no data were collected in low-resource settings, and individuals were not included on the basis of being not responsive to haloperidol as first-line treatment. Moreover, many of the included studies had small sample sizes and high attrition rates.

In conclusion, the clinical implication was that it is not possible to identify individual FGAs to recommend when a first-line treatment with haloperidol proves to be ineffective. Common clinical practice would suggest switching to the other most commonly available treatment or chlorpromazine. However, there is still uncertainty and further studies in low-resources/humanitarian settings may provide relevant insights on this issue.

The therapeutic dose of haloperidol may vary between 2 and 12 mg/day, and a maintenance dose of 4 to 6 mg/day is usually required [35, 37, 38]. For chlorpromazine, the dose range is from 25 to 1000 mg/day, with a maintenance dose of 75 to 300 mg/day [29, 35].

### Are SGAs effective and safe in people with non-affective psychosis who do not improve with FGAs used as first-line treatment?

Each SGA was compared initially to haloperidol, as the reference standard of first-line FGAs. SGAs that proved to be more effective than haloperidol were then compared to each other (head-to-head) in order to identify possible advantages of one medication over another. We found no data specifically referring to individuals who had already failed to improve after treatment with one FGA. Therefore, studies conducted in the general population of people suffering from non-affective psychosis were used (indirect). Compared to haloperidol, all SGAs considered individually (with the exception of paliperidone, for which no data were available) appeared to cause less sedation and motor symptoms, and had a better overall acceptability profile. Among these medications, only risperidone and olanzapine showed a more favourable efficacy profile in comparison with haloperidol. After having compared these two medications head-to-head, risperidone showed better acceptability and was associated with less weight gain than olanzapine, while olanzapine caused less motor symptoms and prolactin increase.

Indirectness, high attrition rates and imprecise results (due to small sample sizes) contributed to set the quality of the evidence to ‘low’ or ‘very low’ for all of the outcomes of interest. In conclusion, the clinical implication was that risperidone and olanzapine may be chosen as second-line treatments as they are both associated with benefits in comparison with haloperidol in terms of efficacy and overall acceptability in the medium- and long-term. The level of confidence in this clinical implication was judged uncertain. The panel pointed out that the choice between risperidone and olanzapine should be based on specific patient characteristics and the antipsychotic profile, considering that, in general, risperidone may have a slightly better overall tolerability and cause less weight gain compared to olanzapine, while olanzapine is associated with less motor symptoms and prolactin increase compared to risperidone. Further, the choice should take into account availability, affordability and sustainability of provision in the long term, in relation to the setting of care. The therapeutic dose of risperidone may vary between 2 and 10 mg/day, and a maintenance dose of 4 to 6 mg/day is usually required [35, 39], while the dose of olanzapine may vary between 5 and 20 mg/day, and the maintenance dose required is usually 10 mg/day [35].

### Which individual antipsychotic is effective and safe in people with a diagnosis of treatment-resistant non-affective psychosis?

For the purposes of this review, individuals were defined as treatment-resistant when at least two adequate trials with different antipsychotics, one of which is a SGA, proved ineffective. This is an adaptation of the definition provided by Suzuki et al. [40]. However, considering the lack of a widely shared consensus on definitions of treatment resistance [41], we included studies of participants with treatment-resistant psychosis even where this definition varied. Compared to FGAs, clozapine appeared to be more effective in terms of clinical improvement, similarly effective in terms of relapse rates, and similarly acceptable and more tolerable in terms of motor symptoms, while blood problems and weight gain were more frequent in patients taking clozapine. In patients with treatment-resistant psychosis, risperidone and olanzapine appeared to be similarly effective and acceptable in comparison with clozapine. Risperidone was associated with less weight gain and sedation, but more motor symptoms, when compared to olanzapine.

Studies comparing clozapine and olanzapine, as well as studies comparing clozapine and risperidone, provided efficacy outcomes of ‘moderate’ quality. By contrast, for most of the other outcomes the quality was ‘low’ or ‘very low’ due to indirectness and high attrition rates.

As a clinical implication, it was concluded that, in patients not improving after at least two antipsychotics (one of which is an SGA) administered at adequate dose and duration, a switch to risperidone or olanzapine (in people with no previous ineffective exposure to these medications) or clozapine may be considered. Almost no evidence exists on other SGAs. The choice to use clozapine must take into account context-related issues, particularly in relation to safety, given that routine clinical and laboratory monitoring (for the risk of life-threatening agranulocytosis, but also for other potentially severe adverse events such as seizures and myocarditis [42]) and supervision by a specialist should be regarded as a fundamental prerequisite.

The therapeutic dose of clozapine may vary between 25 and 900 mg/day, and a maintenance dose of 300 to 600 mg/day is usually required [35, 40, 43].

### Are antidepressant-antipsychotic combinations effective and safe in people with non-affective psychosis who develop depressive, cognitive and negative symptoms?

Augmentation strategies of antipsychotic treatment with antidepressants are often considered in the treatment of depressive, cognitive and negative symptoms in people with non-affective psychosis. These symptom dimensions are often overlooked and may be associated with unfavourable outcomes, such as chronic functional impairment and higher suicide risk [44–46]. We therefore retrieved and analysed all available data on augmentation strategies of antipsychotic treatment with antidepressants.

Adding antidepressants to antipsychotic treatment appeared to be similarly acceptable in comparison with antipsychotics alone, and associated with a statistically relevant benefit on depressive, cognitive and negative symptoms. A ‘low’ and ‘very low’ quality rating was given for all outcomes of interest, due to indirectness, high attrition rates, very small sample sizes and few events for the majority of included studies, which led to imprecise results (Additional file 1).

In conclusion, the clinical implication was that adding one antidepressant to antipsychotic treatment may be considered in the case of clinically relevant depressive, negative or cognitive symptoms. Preference should be given to selective serotonin reuptake inhibitors, including fluoxetine (available widely in generic formulations and included in the WHO essential list of medicines), considering their favourable balance between efficacy and tolerability.

## Discussion

### Quality shortcomings and implications for research

Translating scientific data on antipsychotics into pragmatic suggestions to be implemented in humanitarian settings carries methodological limitations. For this particular setting, indirectness represented the most pressing quality issue. First, none of the studies included in selected reviews were performed in low-resource settings or in humanitarian contexts (Additional file 1). Data were collected in people from stable, high-income, Western countries, which are often considerably different from humanitarian settings with regards to distribution of risks and mediating factors such as medical conditions (e.g. dehydration, malnutrition, infectious illnesses), exposure to potentially traumatic events, stability of family and social support, and access to complementary healthcare resources (e.g. psychosocial support, rehabilitation and a safe medical environment in case of acute symptoms). Second, studies from high-income, Western countries may not capture culturally specific concepts of distress that are relevant to other countries. In general, it is unclear whether the efficacy of treatments may differ in contexts characterised by on-going, chronic adversities [47–49]. Third, even when supported by sound scientific evidence, some interventions may not be feasible in low-resource settings due to the need for expensive and/or time-consuming practices (e.g. laboratory investigations and specialist oversight needed for clozapine). Fourth, the setting may strongly affect the burden and impact of side effects and adverse events. For example, prolactin increase can be managed with relatively sophisticated interventions in high-income countries, including laboratory monitoring of blood prolactin levels, addition of low doses of aripiprazole to the current antipsychotic medication, or the addition of bromocriptine or cabergoline under specialist supervision [50]. None of these options is likely to be feasible and suitable in humanitarian settings. Finally, focusing on the aim of pragmatically translating available data into a clinical guidance, we included reviews referring to populations for which some degree of heterogeneity cannot be excluded, as in the case of treatment-resistant psychosis.

In general, the overall quality of evidence was low for the majority of outcomes considered. This was due not only to the setting of care (as in the case of indirectness), but also resulted from the internal quality of the included studies. Most studies included small samples and had high attrition rates and a short follow-up period, resulting in both inconsistency of estimates across studies and imprecise estimates (even after the aggregation of results from single studies).

For all these reasons, we urgently need to broaden the evidence base around antipsychotic treatments and include direct evidence from populations in low- and middle-income countries and, where possible, from humanitarian settings, instead of merely extrapolating results from studies in high-income settings [51, 52]. Within the scope of this paper, we see a need for pragmatic research to establish the cost-effectiveness of various SGAs and clozapine.

Given the specific challenges related to conducting research in humanitarian settings, such studies should ideally have the form of randomised pragmatic trials focused on issues such as feasibility and cost-effectiveness [53, 54]. This review suggests that the introduction of clozapine as a third step in a treatment protocol could yield significant benefits. However, there is considerable uncertainty about the routine use of clozapine in low- and middle-income countries, mostly because of the risk of serious, and sometimes life-threatening, side effects [55, 56].

### Implications for practice and policy

In recent years, strong emphasis has been placed on addressing mental health issues in low-resource and humanitarian contexts in a timely manner, not only to improve the quality of life for people suffering from mental illness, but also as a necessary step to achieve global health and development goals [57, 58]. The growing number of humanitarian and emergency settings, many of which develop into protracted crises that take years if not decades to resolve, should prompt global health researchers to explicitly take these settings into consideration. The proposed algorithm (Fig. 1) is aimed at optimising not only the quality of treatments, but also the timeliness of care for people with non-affective psychosis, considering that early intervention is widely recognised as an essential precondition for achieving higher response rates and better functioning outcomes [59]. This algorithm shows relevant differences if compared with current guidelines developed for general settings of care in high-income countries [60–63], which confirms how, starting from the same evidence base, clinical decisions can radically differ in relation to context-related preferences, values, feasibility and cost-effectiveness considerations.

Among possible obstacles preventing this guidance from having an effective impact, it should be acknowledged that accurate psychiatric assessment and differential diagnosis might be particularly challenging for non-specialised health workers, especially in emergency and humanitarian contexts. This is generally true for mental health, but particularly relevant for the area of psychosis. For example, the onset of non-affective psychosis is often preceded by non-specific symptoms, such as perplexity, obsessive-compulsive manifestations and sub-threshold mood alterations, whose recognition is challenging even for trained psychiatrists [64]. In addition, in contexts with high levels of disorders related to extreme stress, pre-psychotic stages can be misdiagnosed as mood disorders. At the same time, severe mood or post-traumatic disorders with psychotic features can be mistaken for prodromal psychosis. This is particularly relevant if we consider that culturally related manifestations often include mood episodes with psychotic features [65–67]. Furthermore, the most appropriate options for an effective implementation of mental health recommendations into clinical practice remain unclear [68], and this issue results particularly challenging for humanitarian, low-resources settings [69, 70]. Therefore, although guidance on psychopharmacology can notably improve the effectiveness and timeliness of interventions in humanitarian settings, this tool alone cannot be considered as exhaustive. Sustainable improvements in the quality of treatments for people with severe mental illness need to be supported by a broader cultural and structural change in health systems on multiple levels [71–73].

## Conclusions

Underdiagnosed and undertreated non-affective psychosis is a compelling issue for health workers in humanitarian and emergency settings. By conducting an appraisal of the best evidence base, the present UNHCR guidance attempts to pragmatically address this treatment gap. The adaptation of available scientific evidence to inform clinical practice in humanitarian settings has proved particularly challenging mainly due to indirectness of data, which needs to be urgently supplemented by large and pragmatic in-field clinical research. Hopefully, the implementation of this pragmatic guidance may notably improve cost-effectiveness and timeliness of pharmacological interventions in the context of wide and multilevel actions towards better practices and policies for people with psychosis.

## Additional file

Additional file 1: Online supplemental material - PICO tables and GRADE tables produced for each scoping question. (DOCX 169 kb)

## Abbreviations

FGAs: First-generation antipsychotics
GRADE: Grading of recommendations, assessment, development and evaluation
mhGAP-HIG: Mental Health Gap Action Programme Humanitarian Intervention Guide
mhGAP-IG: Mental Health Gap Action Programme Intervention Guide
SGAs: Second-generation antipsychotics
UNHCR: United Nations High Commissioner for Refugees
WHO: World Health Organization
