# Supplementary material for: Mapping the evidence on pharmacological interventions for non-affective psychosis in humanitarian non-specialised settings: a UNHCR clinical guidance
Source: BMC Med. 2017 Dec 11;15:197. doi: 10.1186/s12916-017-0960-z (PMC5724240; doi:10.1186/s12916-017-0960-z)
Supplement: Additional file 1: — Online supplemental material - PICO tables and GRADE tables produced for each scoping question. (DOCX 169 kb) [file 12916_2017_960_MOESM1_ESM.docx]

**Online supplemental material – PICO tables and GRADE tables produced for each scoping question**

**(1) In people with psychosis who do not improve after treatment with a first-generation antipsychotic (FGA), is switching to another FGA effective and safe?**

**Population/Intervention(s)/Comparator/Outcome(s) (PICO)**

- Population: people suffering from psychosis (including schizophrenia) who failed to improve after treatment with one FGA
- Intervention: haloperidol
- Comparison: chlorpromazine, perphenazine, pimozide, fluphenazine, trifluoperazine and FGAs as a class
- Outcomes:
- Treatment response
- Remission
- Symptoms severity
- Functioning
- Quality of life
- Acceptability
- Tolerability
- Adverse events: endocrine (prolactin increase), motor symptoms, weight gain, sedation
- Setting: humanitarian, low-resources settings

**List of systematic reviews identified by the search process**

*INCLUDED IN GRADE TABLES OR FOOTNOTES*

Tardy M, Huhn M, Kissling W, Engel RR, Leucht S. Haloperidol versus low-potency first-generation antipsychotic medications for schizophrenia. Cochrane Database of Systematic Reviews 2014, Issue 7. Art. No.: CD009268.

Leucht C, Kitzmantel M, Kane J, Leucht S, Chua WLLC. Haloperidol versus chlorpromazine for schizophrenia. Cochrane Database of Systematic Reviews 2008, Issue 1. Art. No.: CD004278.

Hartung B, Sampson S, Leucht S. Perphenazine for schizophrenia. Cochrane Database of Systematic Reviews 2015, Issue 3. Art. No.: CD003443.

Dold M, Samara MT, Li C, Tardy M, Leucht S. Haloperidol versus first-generation antipsychotics for the treatment of schizophrenia and other psychotic disorders. Cochrane Database of Systematic Reviews 2015, Issue 1. Art. No.: CD009831.

**PICO table**

| **Serial n.** | **Intervention/comparison** | **Outcomes** | **Systematic reviews used for GRADE** | **Explanation** |
| --- | --- | --- | --- | --- |
| **I** | haloperidol/chlorpromazine | Treatment response  Remission  Symptoms severity  Functioning  Quality of life  Acceptability  Tolerability  Adverse events:   - endocrine (prolactin) - motor symptoms - weight gain - sedation | Tardy et al., 2008  Data not available  Tardy et al., 2008  Data not available  Data not available  Leucht et al., 2008  Leucht et al., 2008  Leucht et al., 2008  Leucht et al., 2008  Leucht et al., 2008  Leucht et al., 2008 | Note: data from the general population of people with psychosis (indirectness) |
| **II** | haloperidol/perphenazine | Treatment response  Remission  Symptoms severity  Functioning  Quality of life  Acceptability  Tolerability  Adverse events:   - endocrine (prolactin) - motor symptoms - weight gain   sedation | Dold et al., 2015  No data available  Hartung et al., 2015  No data available  No data available  Hartung et al., 2015  Hartung et al., 2015  No data available  Hartung et al., 2015  No data available  No data available | Note: data from the general population of people with psychosis (indirectness) |
| **III** | haloperidol/pimozide | Treatment response  Remission  Symptoms severity  Functioning  Quality of life  Acceptability  Tolerability  Adverse events:   - endocrine (prolactin) - motor symptoms - weight gain   sedation | Dold et al., 2015  No data available  No data available  No data available  No data available  No data available  No data available  No data available  No data available  No data available  No data available | Note: data from the general population of people with psychosis (indirectness) |
| **IV** | haloperidol/fluphenazine | Treatment response  Remission  Symptoms severity  Functioning  Quality of life  Acceptability  Tolerability  Adverse events:   - endocrine (prolactin) - motor symptoms - weight gain   sedation | Dold et al., 2015  No data available  No data available  No data available  No data available  No data available  No data available  No data available  No data available  No data available  No data available | Note: data from the general population of people with psychosis (indirectness) |
| **V** | haloperidol/trifluoperazine | Treatment response  Remission  Symptoms severity  Functioning  Quality of life  Acceptability  Tolerability  Adverse events:   - endocrine (prolactin) - motor symptoms - weight gain   sedation | Dold et al., 2015  No data available  No data available  No data available  No data available  No data available  No data available  No data available  No data available  No data available  No data available | Note: data from the general population of people with psychosis (indirectness) |
| **VI** | haloperidol/FGAs (as a class) | Treatment response  Remission  Symptoms severity  Functioning  Quality of life  Acceptability  Tolerability  Adverse events:   - endocrine (prolactin) - motor symptoms - weight gain   sedation | Dold et al., 2015  No data available  Dold et al., 2015  No data available  No data available  Dold et al., 2015  Dold et al., 2015  No data available  Dold et al., 2015  Dold et al., 2015  Dold et al., 2015 | Note: data from the general population of people with psychosis (indirectness) |

**GRADE tables**

**Author(s)**: Giovanni Ostuzzi

**Date**: 27/01/2016

**Question**: Haloperidol compared to chlorpromazine for people suffering from psychosis (including schizophrenia) who failed to improve after treatment with one FGA

**Setting**: humanitarian/low resource setting

**Bibliography**: Tardy M, Huhn M, Kissling W, Engel RR, Leucht S. Haloperidol versus low-potency first-generation antipsychotic medications for schizophrenia. Cochrane Database of Systematic Reviews 2014, Issue 7. Art. No.: CD009268.

Leucht C, Kitzmantel M, Kane J, Leucht S, Chua WLLC. Haloperidol versus chlorpromazine for schizophrenia. Cochrane Database of Systematic Reviews 2008, Issue 1. Art. No.: CD004278.

| **Quality assessment** | | | | | | | **№ of patients** | | **Effect** | | **Quality** | **Importance** |
| --- | --- | --- | --- | --- | --- | --- | --- | --- | --- | --- | --- | --- |
| **№ of studies** | **Study design** | **Risk of bias** | **Inconsistency** | **Indirectness** | **Imprecision** | **Other considerations** | **haloperidol** | **chlorpromazine** | **Relative (95% CI)** | **Absolute (95% CI)** |  |  |
| Treatment response (short term) (assessed with: 50% reduction on PANNS or BPRS) | | | | | | | | | | | | |
| 7 | randomised trials ^1^ | serious ^2^ | not serious ^3^ | serious ^4^ | not serious ^5^ | publication bias strongly suspected ^6^ | 56/144 (38.9%) | 40/146 (27.4%) | **RR 1.31** (0.97 to 1.77) | **85 more per 1000** (from 8 fewer to 211 more) | ⨁◯◯◯ VERY LOW | CRITICAL |
| Symptoms severity (follow up: mean 4 weeks; assessed with: average endpoint score on BPRS. Lower scores indicate less severe psychopathology; negative values of the MD favour haloperidol; Scale from: 18 to 126) | | | | | | | | | | | | |
| 1 | randomised trials ^7^ | very serious ^8^ | not serious | serious ^4^ | very serious ^9^ | none | 17 | 20 | - | MD **2.7 lower** (7.28 lower to 1.88 higher) | ⨁◯◯◯ VERY LOW | CRITICAL |
| Acceptability (follow up: range 4 weeks to 12 weeks; assessed with: n. of patients leaving the study early for any reason) | | | | | | | | | | | | |
| 8 | randomised trials ^7^ | not serious ^10^ | not serious ^3^ | serious ^4^ | not serious ^11^ | none | 1/167 (0.6%) | 8/116 (6.9%) | **RR 0.15** (0.04 to 0.64) | **59 fewer per 1000** (from 25 fewer to 66 fewer) | ⨁⨁⨁◯ MODERATE | CRITICAL |
| Tolerability (follow up: range 4 weeks to 12 weeks; assessed with: n. of patients leaving the study early due to adverse events) | | | | | | | | | | | | |
| 8 | randomised trials ^7^ | not serious ^10^ | not serious ^3^ | serious ^4^ | not serious ^12^ | none | 0/124 (0.0%) | 5/129 (3.9%) | **RR 0.23** (0.04 to 1.32) | **30 fewer per 1000** (from 12 more to 37 fewer) | ⨁⨁⨁◯ MODERATE | IMPORTANT |
| Sedation (follow up: range 4 weeks to 12 weeks; assessed with: n. of patients experiencing sedation) | | | | | | | | | | | | |
| 3 | randomised trials ^7^ | not serious ^10^ | very serious ^13^ | serious ^4^ | very serious ^14^ | none | 9/43 (20.9%) | 17/43 (39.5%) | **RR 0.35** (0.02 to 5.73) | **257 fewer per 1000** (from 387 fewer to 1000 more) | ⨁◯◯◯ VERY LOW | IMPORTANT |
| Prolactin increase (follow up: range 4 weeks to 12 weeks; assessed with: n. of patients with prolactin increased) | | | | | | | | | | | | |
| 1 | randomised trials ^7^ | not serious ^10^ | not serious | serious ^4^ | serious ^15^ | none | 17/17 (100.0%) | 17/20 (85.0%) | **RR 1.17** (0.95 to 1.43) | **144 more per 1000** (from 43 fewer to 365 more) | ⨁⨁◯◯ LOW | IMPORTANT |
| Weight gain (follow up: range 4 weeks to 12 weeks; assessed with: n. of patients experiencing weight gain) | | | | | | | | | | | | |
| 2 | randomised trials ^7^ | not serious ^10^ | not serious | serious ^4^ | very serious ^16^ | none | 0/24 (0.0%) | 4/24 (16.7%) | **RR 0.11** (0.01 to 1.89) | **148 fewer per 1000** (from 148 more to 165 fewer) | ⨁◯◯◯ VERY LOW | IMPORTANT |
| Motor symptoms (follow up: range 4 weeks to 12 weeks; assessed with: n. of patients using antiparkinson medication) | | | | | | | | | | | | |
| 1 | randomised trials ^7^ | not serious ^10^ | not serious | serious ^4^ | very serious ^17^ | none | 3/10 (30.0%) | 1/10 (10.0%) | **RR 3.00** (0.37 to 24.17) | **200 more per 1000** (from 63 fewer to 1000 more) | ⨁◯◯◯ VERY LOW | IMPORTANT |

**CI:** Confidence interval; **RR:** Risk ratio; **MD:** Mean difference

1. Data from Tardy et al., 2014
2. Only for 3 studies out of 7 the outcome assessment was clearly masked. For 4 studies out of 7 the dropout rate was clearly lower than 30%, whilst for the other 3 studies no sufficient data were provided.
3. I-squared=0%
4. None of the included trials was performed in a low-resources humanitarian setting. Further, data are from the general population of people suffering from psychosis rather than people not responding to a first-line FGA, as this was the best available evidence, although fairly approximate.
5. The number of included individuals is more than 290. The CI doesn’t show a relevant effect and doesn't include appreciable benefit or appreciable harm: CI 95% 1.31 (0.97 to 1.77).
6. Funnel plot strongly asymmetrical.
7. Data from Leucht et al., 2008
8. The included trial was probably open and the dropout rate was not reported.
9. The number of individuals included was 37 and the 95% CI crosses both no effect and appreciable benefit (about 7 point decrease on BPRS) for haloperidol.
10. For most studies it is not clear whether the assessment was masked or not, however this is likely to be a problem of reporting rather than a true detection bias. Dropout rates are relatively low for each included study.
11. The number of individuals included is 283, the 95% CI is narrow.
12. The number of individuals included is 253, the 95% CI is narrow.
13. I-squared=85%
14. The overall number of individuals enrolled is 86. The 95% CI crosses one (no effect) and both 0.5 (appreciable harm) and 2 (appreciable benefit).
15. The overall number of individuals enrolled is 37. The 95% CI is relatively narrow.
16. The overall number of individuals enrolled is 48. The 95% CI crosses one (no effect) and 0.5 (appreciable harm).
17. The overall number of individuals enrolled is 20. The 95% CI crosses one (no effect) and both 0.5 (appreciable benefit) and 2 (appreciable harm).

**Author(s)**: Giovanni Ostuzzi

**Date**: 27/01/2016

**Question**: Perphenazine compared to haloperidol for people suffering from psychosis (including schizophrenia) who failed to improve after treatment with one FGA

**Setting**: humanitarian/low resource setting

**Bibliography**: Hartung B, Sampson S, Leucht S. Perphenazine for schizophrenia. Cochrane Database of Systematic Reviews 2015, Issue 3. Art. No.: CD003443.

Dold M, Samara MT, Li C, Tardy M, Leucht S. Haloperidol versus first-generation antipsychotics for the treatment of schizophrenia and other psychotic disorders. Cochrane Database of Systematic Reviews 2015, Issue 1. Art. No.: CD009831.

| **Quality assessment** | | | | | | | **№ of patients** | | **Effect** | | **Quality** | **Importance** |
| --- | --- | --- | --- | --- | --- | --- | --- | --- | --- | --- | --- | --- |
| **№ of studies** | **Study design** | **Risk of bias** | **Inconsistency** | **Indirectness** | **Imprecision** | **Other considerations** | **perphenazine** | **haloperidol** | **Relative (95% CI)** | **Absolute (95% CI)** |  |  |
| Treatment response (short term) (assessed with: 50% reduction on PANNS or BPRS) | | | | | | | | | | | | |
| 1 | randomised trials ^1^ | serious ^2^ | not serious | serious ^3^ | very serious ^4^ | none | 9/18 (50.0%) | 15/21 (71.4%) | **RR 0.70** (0.41 to 1.20) | **214 fewer per 1000** (from 143 more to 421 fewer) | ⨁◯◯◯ VERY LOW | CRITICAL |
| Symptoms severity (short term) (assessed with: change over time - no better or deterioration - ITT) | | | | | | | | | | | | |
| 1 | randomised trials ^5^ | not serious ^6^ | not serious | serious ^3^ | not serious ^7^ | none | 55/95 (57.9%) | 54/94 (57.4%) | **RR 1.01** (0.79 to 1.29) | **6 more per 1000** (from 121 fewer to 167 more) | ⨁⨁⨁◯ MODERATE | CRITICAL |
| Acceptability (short term) (assessed with: n. of patients leaving the study early due to any reason) | | | | | | | | | | | | |
| 1 | randomised trials ^5^ | serious ^8^ | not serious | serious ^3^ | serious ^9^ | none | 11/95 (11.6%) | 13/94 (13.8%) | **RR 0.84** (0.40 to 1.77) | **22 fewer per 1000** (from 83 fewer to 106 more) | ⨁◯◯◯ VERY LOW | CRITICAL |
| Tolerability (short term) (assessed with: n. of patients leaving the study early due to adverse events) | | | | | | | | | | | | |
| 1 | randomised trials ^5^ | serious ^8^ | not serious | serious ^3^ | serious ^9^ | none | 3/95 (3.2%) | 6/94 (6.4%) | **RR 0.49** (0.13 to 1.92) | **33 fewer per 1000** (from 56 fewer to 59 more) | ⨁◯◯◯ VERY LOW | IMPORTANT |
| Movement disorders (short term) (assessed with: use of antiparkinsonian medications) | | | | | | | | | | | | |
| 1 | randomised trials ^5^ | serious ^8^ | not serious | serious ^3^ | serious ^9^ | none | 51/95 (53.7%) | 58/94 (61.7%) | **RR 0.87** (0.68 to 1.11) | **80 fewer per 1000** (from 68 more to 197 fewer) | ⨁◯◯◯ VERY LOW | IMPORTANT |

**CI:** Confidence interval; **RR:** Risk ratio

1. From Dold et al., 2015.
2. In the only study included 15 of 75 participants (20%) left the trial early.
3. None of the included trials was performed in a low-resources humanitarian setting. Further, data are from the general population of people suffering from psychosis rather than people not responding to a first-line FGA, as this was the best available evidence, although fairly approximate.
4. The study enrolled 39 patients and the 95% CI crosses both one (no effect) and 0.5 (appreciable harm).
5. From Hartung et al., 2015.
6. The dropout rate was lower than 30%. No clear information on assessment masking, however this is likely to be a problem of reporting rather than a true detection bias.
7. The study included 99 individuals. The 95% CI is relatively narrow.
8. The dropout rate and the outcome assessment are not clearly described for the only included trial.
9. The included study enrolled 189 patients and the 95% CI interval crosses both one (no effect) and 0.5 (appreciable harm).

**Author(s)**: Giovanni Ostuzzi

**Date:** 27/01/2016

**Question**: Pimozide compared to haloperidol for people suffering from psychosis (including schizophrenia) who failed to improve after treatment with one FGA

**Setting**: humanitarian/low resource setting

**Bibliography**: Dold M, Samara MT, Li C, Tardy M, Leucht S. Haloperidol versus first-generation antipsychotics for the treatment of schizophrenia and other psychotic disorders. Cochrane Database of Systematic Reviews 2015, Issue 1. Art. No.: CD009831.

| **Quality assessment** | | | | | | | **№ of patients** | | **Effect** | | **Quality** | **Importance** |
| --- | --- | --- | --- | --- | --- | --- | --- | --- | --- | --- | --- | --- |
| **№ of studies** | **Study design** | **Risk of bias** | **Inconsistency** | **Indirectness** | **Imprecision** | **Other considerations** | **pimozide** | **haloperidol** | **Relative (95% CI)** | **Absolute (95% CI)** |  |  |
| Treatment response (short term) (assessed with: 50% reduction on PANNS or BPRS) | | | | | | | | | | | | |
| 3 | randomized trials | not serious | not serious ^1^ | serious ^2^ | very serious ^3^ | none | 20/35 (57.1%) | 23/37 (62.2%) | **RR 1.09** (0.74 to 1.61) | **56 more per 1000** (from 162 fewer to 379 more) | ⨁◯◯◯ VERY LOW | CRITICAL |

**CI:** Confidence interval; **RR:** Risk ratio

1. I-squared=3%
2. None of the included trials was performed in a low-resources humanitarian setting. Further, data are from the general population of people suffering from psychosis rather than people not responding to a first-line FGA, as this was the best available evidence, although fairly approximate.
3. The overall number of patients enrolled in the three included trials was 72. The 95% CI crosses one (no effect) but not 0.5 (appreciable harm).

**Author(s)**: Giovanni Ostuzzi

**Date**: 27/01/2016

**Question**: Fluphenazine compared to haloperidol for people suffering from psychosis (including schizophrenia) who failed to improve after treatment with one FGA

**Setting**: humanitarian/low resources setting

**Bibliography**: Dold M, Samara MT, Li C, Tardy M, Leucht S. Haloperidol versus first-generation antipsychotics for the treatment of schizophrenia and other psychotic disorders. Cochrane Database of Systematic Reviews 2015, Issue 1. Art. No.: CD009831.

| **Quality assessment** | | | | | | | **№ of patients** | | **Effect** | | **Quality** | **Importance** |
| --- | --- | --- | --- | --- | --- | --- | --- | --- | --- | --- | --- | --- |
| **№ of studies** | **Study design** | **Risk of bias** | **Inconsistency** | **Indirectness** | **Imprecision** | **Other considerations** | **fluphenazine** | **haloperidol** | **Relative (95% CI)** | **Absolute (95% CI)** |  |  |
| Treatment response (short term) (assessed with: 50% reduction on PANNS or BPRS) | | | | | | | | | | | | |
| 3 | randomized trials | not serious ^1^ | not serious ^2^ | serious ^3^ | serious ^4^ | none | 13/89 (14.6%) | 9/68 (13.2%) | **RR 0.78** (0.37 to 1.65) | **29 fewer per 1000** (from 83 fewer to 86 more) | ⨁⨁◯◯ LOW | CRITICAL |

**CI:** Confidence interval; **RR:** Risk ratio

1. For two studies the dropout rate was lower than 30% and for one study the dropout rate was not reported. The assessment masking was not clearly reported.
2. I-squared=0%
3. None of the included trials was performed in low-resources settings. Further, data are from the general population of people suffering from psychosis rather than people not responding to a first-line FGA, as this was the best available evidence, although fairly approximate.
4. The three trials included 157 patients overall. The 95% CI crosses one (no effect) and 0.5 (appreciable harm).

**Author(s)**: Giovanni Ostuzzi

**Date**: 27/01/2016

**Question**: Trifluoperazine compared to haloperidol for people suffering from psychosis (including schizophrenia) who failed to improve after treatment with one FGA

**Setting**: humanitarian/low resources setting

**Bibliography**: Dold M, Samara MT, Li C, Tardy M, Leucht S. Haloperidol versus first-generation antipsychotics for the treatment of schizophrenia and other psychotic disorders. Cochrane Database of Systematic Reviews 2015, Issue 1. Art. No.: CD009831.

| **Quality assessment** | | | | | | | **№ of patients** | | **Effect** | | **Quality** | **Importance** |
| --- | --- | --- | --- | --- | --- | --- | --- | --- | --- | --- | --- | --- |
| **№ of studies** | **Study design** | **Risk of bias** | **Inconsistency** | **Indirectness** | **Imprecision** | **Other considerations** | **trifluoperazine** | **haloperidol** | **Relative (95% CI)** | **Absolute (95% CI)** |  |  |
| Treatment response (short term) (assessed with: 50% reduction on PANNS or BPRS) | | | | | | | | | | | | |
| 4 | randomized trials | not serious ^1^ | not serious ^2^ | serious ^3^ | serious ^4^ | none | 26/61 (42.6%) | 27/63 (42.9%) | **RR 1.01** (0.68 to 1.50) | **4 more per 1000** (from 137 fewer to 214 more) | ⨁⨁◯◯ LOW | CRITICAL |

**CI:** Confidence interval; **RR:** Risk ratio

1. For one study the dropout rate was higher than 30% and for one study the dropout rate was not reported. For none of the included studies it was clearly reported whether the assessment was masked or not.
2. I-squared=0%
3. None of the included trials was performed in low-resources settings. Further, data are from the general population of people suffering from psychosis rather than people not responding to a first-line FGA, as this was the best available evidence, although fairly approximate.
4. The four included studies enrolled 124 patients overall. The 95% CI is relatively narrow.

**Author(s)**: Giovanni Ostuzzi

**Date**: 27/01/2016

**Question**: Haloperidol compared to other FGAs for people suffering from psychosis (including schizophrenia) who failed to improve after treatment with one FGA

**Setting**: humanitarian/low resources setting

**Bibliography**: Dold M, Samara MT, Li C, Tardy M, Leucht S. Haloperidol versus first-generation antipsychotics for the treatment of schizophrenia and other psychotic disorders. Cochrane Database of Systematic Reviews 2015, Issue 1. Art. No.: CD009831.

| **Quality assessment** | | | | | | | **№ of patients** | | **Effect** | | **Quality** | **Importance** |
| --- | --- | --- | --- | --- | --- | --- | --- | --- | --- | --- | --- | --- |
| **№ of studies** | **Study design** | **Risk of bias** | **Inconsistency** | **Indirectness** | **Imprecision** | **Other considerations** | **haloperidol** | **other FGAs** | **Relative (95% CI)** | **Absolute (95% CI)** |  |  |
| Treatment response (short term) (assessed with: 50% score reduction on PANNS or BPRS) | | | | | | | | | | | | |
| 40 | randomised trials | not serious ^1^ | not serious ^2^ | serious ^3^ | not serious ^4^ | none | 430/1045 (41.1%) | 497/1087 (45.7%) | **RR 0.93** (0.87 to 1.00) | **32 fewer per 1000** (from 0 fewer to 59 fewer) | ⨁⨁⨁◯ MODERATE | IMPORTANT |
| Symptoms severity (short term) (assessed with: mean BPRS score at endpoint. Lower scores indicate less severe psychopathology; negative values of the MD favour haloperidol; Scale from: 18 to 126) | | | | | | | | | | | | |
| 23 | randomised trials | not serious ^7^ | very serious ^8^ | serious ^3^ | not serious ^9^ | none | 492 | 506 | - | MD **0.37 higher** (1.66 lower to 2.39 higher) | ⨁◯◯◯ VERY LOW | IMPORTANT |
| Acceptability (short term) (assessed with: n. of patients leaving the study early due to any reason) | | | | | | | | | | | | |
| 28 | randomised trials | not serious ^7^ | not serious ^2^ | serious ^3^ | not serious ^10^ | none | 138/653 (21.1%) | 130/646 (20.1%) | **RR 1.04** (0.87 to 1.24) | **8 more per 1000** (from 26 fewer to 48 more) | ⨁⨁⨁◯ MODERATE | IMPORTANT |
| Tolerability (short term) (assessed with: n. of patients leaving the study early due to adverse events) | | | | | | | | | | | | |
| 16 | randomised trials | not serious ^7^ | not serious ^2^ | serious ^3^ | serious ^11^ | none | 10/319 (3.1%) | 10/321 (3.1%) | **RR 1.00** (0.42 to 2.35) | **0 fewer per 1000** (from 18 fewer to 42 more) | ⨁⨁◯◯ LOW | IMPORTANT |
| Motor symptoms (short term) (assessed with: n. of patients experiencing at least one extrapyramidal/movement disorder) | | | | | | | | | | | | |
| 17 | randomised trials | not serious ^7^ | not serious ^12^ | serious ^3^ | not serious ^9^ | none | 211/500 (42.2%) | 182/498 (36.5%) | **RR 1.12** (0.95 to 1.31) | **44 more per 1000** (from 18 fewer to 113 more) | ⨁⨁⨁◯ MODERATE | IMPORTANT |
| Sedation (short term) (assessed with: n. of patients experiencing sedation) | | | | | | | | | | | | |
| 5 | randomised trials | not serious ^13^ | not serious ^14^ | serious ^3^ | not serious ^15^ | none | 28/158 (17.7%) | 41/152 (27.0%) | **RR 0.72** (0.45 to 1.18) | **76 fewer per 1000** (from 49 more to 148 fewer) | ⨁⨁⨁◯ MODERATE | IMPORTANT |
| Weight gain (short term) (assessed with: n. of patients experiencing weight gain) | | | | | | | | | | | | |
| 6 | randomised trials | serious ^16^ | serious ^17^ | serious ^3^ | serious ^18^ | none | 14/129 (10.9%) | 24/133 (18.0%) | **RR 0.67** (0.21 to 2.15) | **60 fewer per 1000** (from 143 fewer to 208 more) | ⨁◯◯◯ VERY LOW | IMPORTANT |

**CI:** Confidence interval; **RR:** Risk ratio; **MD:** Mean difference

1. The authors of this Cochrane review detected a high risk of attrition for only two of the studies included in this analysis. In general, the blinding of assessment was unclearly reported (Fig. 2 and 3, pp. 19-20).
2. I-squared=0%
3. None of the included studies was performed in low-resources/humanitarian settings. Further, data are from the general population of people suffering from psychosis rather than people not responding to a first-line FGA, as this was the best available evidence, although fairly approximate.
4. The analysis included 2132 patients. The 95% CI is narrow.
5. Information about the masking of assessment and the dropout rates are not reported for 2 of the three included studies.
6. 130 patients included in the analysis. The 95% CI crosses both 1 (no effect) and 2 (appreciable benefit).
7. The dropout rate was lower than 30% for the large majority of studies.
8. I-squared=91%
9. The analysis included 998 patients. The 95% CI is narrow.
10. The analysis included 1299 patients. The 95% CI is narrow.
11. The study included 640 patients. The 95% CI crosses both 1 (no effect) and 2 (appreciable benefit).
12. I-squared=17%
13. Four studies over 5 had an attrition rate lower than 30% and for one study this information was not reported.
14. I-squared=75%
15. The analysis included 310 patients. The 95% CI is narrow.
16. For three studies over 6 the attrition rate lower than 30%, for one study this was higher and for two studies this information was not reported.
17. I-squared=58%
18. The study included 262 patients. The 95% CI crosses both 0.5 (appreciable harm) and 2 (appreciable benefit).

**(2) Are second-generation antipsychotics (SGAs) effective and safe in people with psychosis who do not improve with FGAs used as first-line treatment?**

**Population/Intervention(s)/Comparator/Outcome(s) (PICO)**

- Population: people suffering from psychosis (including schizophrenia) who failed to improve after treatment with one FGA
- Intervention: risperidone/olanzapine/quetiapine/aripiprazole/paliperidone
- Comparison: haloperidol
- Outcomes:
- Treatment response
- Remission
- Symptoms severity
- Functioning
- Quality of life
- Acceptability
- Tolerability
- Adverse events: endocrine (prolactin increase), motor symptoms, weight gain, sedation
- Setting: humanitarian, low-resources settings

**List of the systematic reviews identified by the search process**

*INCLUDED IN GRADE TABLES OR FOOTNOTES*

Leucht S, Cipriani A, Spineli L, Mavridis D, Orey D, Richter F, Samara M, Barbui C, Engel RR, Geddes JR, Kissling W, Stapf MP, Lässig B, Salanti G, Davis JM. Comparative efficacy and tolerability of 15 antipsychotic medications in schizophrenia: A multiple-treatments meta-analysis. Lancet 2013;382(9896):951-62.

Komossa K, Rummel-Kluge C, Schwarz S, Schmid F, Hunger H, Kissling W, Leucht S. Risperidone versus other atypical antipsychotics for schizophrenia. Cochrane Database of Systematic Reviews 2011, Issue 1. Art. No.: CD006626.

**PICO table**

| **Serial n.** | **Intervention/comparison** | **Outcomes** | **Systematic reviews used for GRADE** | **Explanation** |
| --- | --- | --- | --- | --- |
| **I** | haloperidol/risperidone | Treatment response  Remission  Symptoms severity  Functioning  Quality of life  Acceptability  Tolerability  Adverse events:   - endocrine (prolactin) - motor symptoms - weight gain - sedation | Data not available  Data not available  Leucth et al., 2013  Data not available  Data not available  Leucth et al., 2013  Data not available  Leucth et al., 2013  Leucth et al., 2013  Leucth et al., 2013  Leucth et al., 2013 | Note: data from the general population of people with psychosis (indirectness) |
| **II** | haloperidol/olanzapine | Treatment response  Remission  Symptoms severity  Functioning  Quality of life  Acceptability  Tolerability  Adverse events:   - endocrine (prolactin) - motor symptoms - weight gain - sedation | Data not available  Data not available  Leucth et al., 2013  Data not available  Data not available  Leucth et al., 2013  Data not available  Leucth et al., 2013  Leucth et al., 2013  Leucth et al., 2013  Leucth et al., 2013 | Note: data from the general population of people with psychosis (indirectness) |
| **III** | haloperidol/quetiapine | Treatment response  Remission  Symptoms severity  Functioning  Quality of life  Acceptability  Tolerability  Adverse events:   - endocrine (prolactin) - motor symptoms - weight gain - sedation | Data not available  Data not available  Leucth et al., 2013  Data not available  Data not available  Leucth et al., 2013  Data not available  Leucth et al., 2013  Leucth et al., 2013  Leucth et al., 2013  Leucth et al., 2013 | Note: data from the general population of people with psychosis (indirectness) |
| **IV** | haloperidol/aripiprazole | Treatment response  Remission  Symptoms severity  Functioning  Quality of life  Acceptability  Tolerability  Adverse events:   - endocrine (prolactin) - motor symptoms - weight gain - sedation | Data not available  Data not available  Leucth et al., 2013  Data not available  Data not available  Leucth et al., 2013  Data not available  Leucth et al., 2013  Leucth et al., 2013  Leucth et al., 2013  Leucth et al., 2013 | Note: data from the general population of people with psychosis (indirectness) |
| **V** | haloperidol/paliperidone | Treatment response  Remission  Symptoms severity  Functioning  Quality of life  Acceptability  Tolerability  Adverse events:   - endocrine (prolactin) - motor symptoms - weight gain - sedation | Data not available  Data not available  Data not available  Data not available  Data not available  Data not available  Data not available  Data not available  Data not available  Data not available  Data not available | Note: data from the general population of people with psychosis (indirectness) |
| **VI** | risperidone/olanzapine | Treatment response  Remission  Symptoms severity  Functioning  Quality of life  Acceptability  Tolerability  Adverse events:   - endocrine (prolactin) - motor symptoms - weight gain - sedation | Komossa et al., 2011  Data not available  Komossa et al., 2011  Data not available  Komossa et al., 2011  Komossa et al., 2011  Komossa et al., 2011  Komossa et al., 2011  Komossa et al., 2011  Komossa et al., 2011  Komossa et al., 2011 | Note: data from the general population of people with psychosis (indirectness) |

**GRADE tables**

**Author(s)**: Giovanni Ostuzzi

**Date**: 27/01/2016

**Question**: Risperidone compared to haloperidol for people suffering from psychosis (including schizophrenia) who failed to improve after treatment with one FGA

**Setting**: humanitarian/low resources setting

**Bibliography**: Leucht S, Cipriani A, Spineli L, Mavridis D, Orey D, Richter F, Samara M, Barbui C, Engel RR, Geddes JR, Kissling W, Stapf MP, Lässig B, Salanti G, Davis JM. Comparative efficacy and tolerability of 15 antipsychotic medications in schizophrenia: a multiple-treatments meta-analysis. Lancet 2013, 14;382(9896):951-62.

| **Quality assessment** | | | | | | | **№ of patients** | | **Effect** | | **Quality** | **Importance** |
| --- | --- | --- | --- | --- | --- | --- | --- | --- | --- | --- | --- | --- |
| **№ of studies** | **Study design** | **Risk of bias** | **Inconsistency** | **Indirectness** | **Imprecision** | **Other considerations** | **risperidone** | **haloperidol** | **Relative (95% CI)** | **Absolute (95% CI)** |  |  |
| Efficacy (follow up: range 4 weeks to 12 weeks; assessed with: PANNS or BPRS total score) | | | | | | | | | | | | |
| 16 | randomised trials ^1^ | serious ^2^ | not serious ^3^ | serious ^4^ | not serious ^5^ | none | 1795 patients included in the analysis. Not clear how many patients were allocated to each arm. The SMD significantly favours risperidone: SMD 0.17, 95% CrI (Credibility Interval) 0.01 to 0.33. | | | | ⨁⨁◯◯ LOW | CRITICAL |
| Acceptability (follow up: range 4 weeks to 12 weeks; assessed with: n. of patients leaving the study early for any reason) | | | | | | | | | | | | |
| 16 | randomised trials ^1^ | serious ^2^ | not serious ^3^ | serious ^4^ | not serious ^5^ | none | 1795 patients included in the analysis. Not clear how many patients were allocated to each arm. The OR significantly favours risperidone: OR 1.54, 95% CrI (Credibility Interval) 1.25 to 1.90. | | | | ⨁⨁◯◯ LOW | CRITICAL |
| Weight gain (follow up: range 4 weeks to 12 weeks; assessed with: weight gain at the study endpoint) | | | | | | | | | | | | |
| 16 | randomised trials ^1^ | serious ^2^ | not serious ^3^ | serious ^4^ | not serious ^5^ | none | 1795 patients included in the analysis. Not clear how many patients were allocated to each arm. The SMD does not show a significant advantage of one medication over the other: SMD -0.15, 95% CrI (Credibility Interval) -0.44 to 0.14 (SMD lower than 0 favours haloperidol). | | | | ⨁⨁◯◯ LOW | IMPORTANT |
| Prolactin (follow up: range 4 weeks to 12 weeks; assessed with: serum concentration at the study endpoint) | | | | | | | | | | | | |
| 16 | randomised trials ^1^ | serious ^2^ | not serious ^3^ | serious ^4^ | not serious ^5^ | none | 1795 patients included in the analysis. Not clear how many patients were allocated to each arm. The SMD is on the edge of significance for haloperidol (suggesting slightly more patients with prolactin increase with risperidone): SMD -0.32, 95% CrI (Credibility Interval) -0.68 to 0.03. | | | | ⨁⨁◯◯ LOW | IMPORTANT |
| Motor symptoms (follow up: range 4 weeks to 12 weeks; assessed with: use of antiparkinson medications) | | | | | | | | | | | | |
| 16 | randomised trials ^1^ | serious ^2^ | not serious ^3^ | serious ^4^ | not serious ^5^ | none | 1795 patients included in the analysis. Not clear how many patients were allocated to each arm. The OR significantly favours risperidone (suggesting fewer patients using antiparkinson medications with risperidone): OR 2.08, 95% CrI (Credibility Interval) 1.54 to 2.82. | | | | ⨁⨁◯◯ LOW | IMPORTANT |
| Sedation (follow up: range 4 weeks to 12 weeks; assessed with: n. of patients experiencing sedation) | | | | | | | | | | | | |
| 16 | randomised trials ^1^ | serious ^2^ | not serious ^3^ | serious ^4^ | not serious ^5^ | none | 1795 patients included in the analysis. Not clear how many patients were allocated to each arm. The OR significantly favours risperidone (suggesting fewer patients experiencing sedation with risperidone): OR 1.20, 95% CrI (Credibility Interval) 0.65 to 2.21. | | | | ⨁⨁◯◯ LOW | IMPORTANT |

**CI:** Confidence interval

1. Only direct comparisons from the Network Meta-analysis by Leucht et al. (2013) were considered.
2. Details for each of the included studies are limited. According to the overall quality of included studies (appendix 6b), details of the assessment masking are lacking in about half of the studies and the risk of attrition bias is high in almost 50% of the studies. However, we cannot rely on the assumption that such trend can be simply extended to the studies included in this specific head-to-head comparison. Thus, we applied a downgrade of -1 instead of -2.
3. Details not available for this particular comparison.
4. None of the included trials was performed in a low-resources humanitarian setting, Further, data are from the general population of people suffering from psychosis rather than people not responding to a first-line FGA, as this was the best available evidence, although fairly approximate.
5. The analysis includes 1795 patients. The 95% CrI is relatively narrow.

**Author(s)**: Giovanni Ostuzzi

**Date**: 27/01/2016

**Question**: Olanzapine compared to haloperidol for people suffering from psychosis (including schizophrenia) who failed to improve after treatment with one FGA

**Setting**: humanitarian/low resources settings

**Bibliography**: Leucht S, Cipriani A, Spineli L, Mavridis D, Orey D, Richter F, Samara M, Barbui C, Engel RR, Geddes JR, Kissling W, Stapf MP, Lässig B, Salanti G, Davis JM. Comparative efficacy and tolerability of 15 antipsychotic medications in schizophrenia: a multiple-treatments meta-analysis. Lancet 2013, 14;382(9896):951-62.

| **Quality assessment** | | | | | | | **№ of patients** | | **Effect** | | **Quality** | **Importance** |
| --- | --- | --- | --- | --- | --- | --- | --- | --- | --- | --- | --- | --- |
| **№ of studies** | **Study design** | **Risk of bias** | **Inconsistency** | **Indirectness** | **Imprecision** | **Other considerations** | **olanzapine** | **haloperidol** | **Relative (95% CI)** | **Absolute (95% CI)** |  |  |
| Efficacy (follow up: range 4 weeks to 12 weeks; assessed with: PANSS or BPRS total score) | | | | | | | | | | | | |
| 11 | randomised trials ^1^ | serious ^2^ | not serious ^3^ | serious ^4^ | not serious ^5^ | none | 3202 patients included in the analysis. Not clear how many patients were allocated to each arm. The SMD significantly favours olanzapine: SMD 0.17, 95% CrI (Credibility Interval) 0.10 to 0.24. | | | | ⨁⨁◯◯ LOW | CRITICAL |
| Acceptability (follow up: range 4 weeks to 12 weeks; assessed with: n. of patients leaving the study early for any reason) | | | | | | | | | | | | |
| 11 | randomised trials ^1^ | serious ^2^ | not serious ^3^ | serious ^4^ | not serious ^5^ | none | 3202 patients included in the analysis. Not clear how many patients were allocated to each arm. The OR significantly favours olanzapine: OR 1.58, 95% CrI (Credibility Interval) 1.18 to 2.12. | | | | ⨁⨁◯◯ LOW | CRITICAL |
| Weight gain (follow up: range 4 weeks to 12 weeks; assessed with: weight gain at the study endpoint) | | | | | | | | | | | | |
| 11 | randomised trials ^1^ | serious ^2^ | not serious ^3^ | serious ^4^ | not serious ^5^ | none | 3202 patients included in the analysis. Not clear how many patients were allocated to each arm. The SMD significantly favours haloperidol: SMD -0.72, 95% CrI (Credibility Interval) -0.96 to -0.48. | | | | ⨁⨁◯◯ LOW | IMPORTANT |
| Prolactin (follow up: range 4 weeks to 12 weeks; assessed with: serum concentration) | | | | | | | | | | | | |
| 11 | randomised trials ^1^ | serious ^2^ | not serious ^3^ | serious ^4^ | not serious ^5^ | none | 3202 patients included in the analysis. Not clear how many patients were allocated to each arm. The SMD significantly favours olanzapine: SMD 0.56, 95% CrI (Credibility Interval) 0.38 to 0.74. | | | | ⨁⨁◯◯ LOW | IMPORTANT |
| Motor symptoms (follow up: range 4 weeks to 12 weeks; assessed with: use of antiparkinson medications) | | | | | | | | | | | | |
| 11 | randomised trials ^1^ | serious ^2^ | not serious ^3^ | serious ^4^ | not serious ^5^ | none | 3202 patients included in the analysis. Not clear how many patients were allocated to each arm. The OR significantly favours olanzapine: OR 4.79, 95 % CrI 3.08 to 7.45. | | | | ⨁⨁◯◯ LOW | IMPORTANT |
| Sedation (follow up: range 4 weeks to 12 weeks; assessed with: n. of patients experiencing sedation) | | | | | | | | | | | | |
| 11 | randomised trials ^1^ | serious ^2^ | not serious ^3^ | serious ^4^ | not serious ^5^ | none | 3202 patients included in the analysis. Not clear how many patients were allocated to each arm. The OR significantly favours olanzapine: OR 1.22, 95 % CrI 1.00 to 1.47. | | | | ⨁⨁◯◯ LOW | IMPORTANT |

**CI:** Confidence interval

1. Only direct comparisons from the Network Meta-analysis by Leucht et al. (2013) were considered.
2. Details for each of the included studies are limited. According to the overall quality of included studies (appendix 6b), details of the assessment masking are lacking in about half of the studies and the risk of attrition bias is high in almost 50% of the studies. However, we cannot rely on the assumption that such trend can be simply extended to the studies included in this specific head-to-head comparison. Thus, we applied a downgrade of -1 instead of -2.
3. Details not available for this particular comparison.
4. None of the included trials was performed in a low-resources humanitarian setting. Further, data are from the general population of people suffering from psychosis rather than people not responding to a first-line FGA, as this was the best available evidence, although fairly approximate.
5. 3202 patients were included in the analysis. The 95% CrI is relatively narrow.

**Author(s)**: Giovanni Ostuzzi

**Date**: 27/01/2016

**Question**: Quetiapine compared to haloperidol for people suffering from psychosis (including schizophrenia) who failed to improve after treatment with one FGA

**Setting**: humanitarian/low-resources setting

**Bibliography**: Leucht S, Cipriani A, Spineli L, Mavridis D, Orey D, Richter F, Samara M, Barbui C, Engel RR, Geddes JR, Kissling W, Stapf MP, Lässig B, Salanti G, Davis JM. Comparative efficacy and tolerability of 15 antipsychotic medications in schizophrenia: a multiple-treatments meta-analysis. Lancet 2013, 14;382(9896):951-62.

| **Quality assessment** | | | | | | | **№ of patients** | | **Effect** | | **Quality** | **Importance** |
| --- | --- | --- | --- | --- | --- | --- | --- | --- | --- | --- | --- | --- |
| **№ of studies** | **Study design** | **Risk of bias** | **Inconsistency** | **Indirectness** | **Imprecision** | **Other considerations** | **quetiapine** | **haloperidol** | **Relative (95% CI)** | **Absolute (95% CI)** |  |  |
| Efficacy (follow up: range 4 weeks to 12 weeks; assessed with: PANNS or BPRS total score) | | | | | | | | | | | | |
| 4 | randomised trials ^1^ | serious ^2^ | not serious ^3^ | serious ^4^ | not serious ^5^ | none | 792 patients included in the analysis. Not clear how many patients were allocated to each arm. The SMD is on the edge of significance in favour of haloperidol: SMD -0.09, 95% CrI (Credibility Interval) -0.23 to 0.06. | | | | ⨁⨁◯◯ LOW | CRITICAL |
| Acceptability (follow up: range 4 weeks to 12 weeks; assessed with: n. of patients leaving the study early for any reason) | | | | | | | | | | | | |
| 4 | randomised trials ^1^ | serious ^2^ | not serious ^3^ | serious ^4^ | not serious ^5^ | none | 792 patients included in the analysis. Not clear how many patients were allocated to each arm. The SMD significantly favours quetiapine: OR 1.72, 95% CrI (Credibility Interval) 1.01 to 2.93. | | | | ⨁⨁◯◯ LOW | CRITICAL |
| Weight gain (follow up: range 4 weeks to 12 weeks; assessed with: weight gain at the study endpoint) | | | | | | | | | | | | |
| 4 | randomised trials ^1^ | serious ^2^ | not serious ^3^ | serious ^4^ | not serious ^5^ | none | 792 patients included in the analysis. Not clear how many patients were allocated to each arm. The SMD significantly favours haloperidol: SMD -0.35, 95% CrI (Credibility Interval) -0.51 to -0.19. | | | | ⨁⨁◯◯ LOW | IMPORTANT |
| Prolactin (follow up: range 4 weeks to 12 weeks; assessed with: serum concentration at the study endpoint) | | | | | | | | | | | | |
| 4 | randomised trials ^1^ | serious ^2^ | not serious ^3^ | serious ^4^ | not serious ^5^ | none | 792 patients included in the analysis. Not clear how many patients were allocated to each arm. The SMD significantly favours quetiapine: SMD 0.52, 95% CrI (Credibility Interval) 0.36 to 0.69. | | | | ⨁⨁◯◯ LOW | IMPORTANT |
| Motor symptoms (follow up: range 4 weeks to 12 weeks; assessed with: use of antiparkinson medications) | | | | | | | | | | | | |
| 4 | randomised trials ^1^ | serious ^2^ | not serious ^3^ | serious ^4^ | not serious ^5^ | none | 792 patients included in the analysis. Not clear how many patients were allocated to each arm. The OR significantly favours quetiapine: OR 5.08, 95% CrI (Credibility Interval) 3.02 to 8.55. | | | | ⨁⨁◯◯ LOW | IMPORTANT |
| Sedation (follow up: range 4 weeks to 12 weeks; assessed with: n. of patients experiencing sedation) | | | | | | | | | | | | |
| 4 | randomised trials ^1^ | serious ^2^ | not serious ^3^ | serious ^4^ | not serious ^5^ | none | 792 patients included in the analysis. Not clear how many patients were allocated to each arm. The OR significantly favours quetiapine: OR 0.79, 95% CrI (Credibility Interval) 0.34 to 1.85. | | | | ⨁⨁◯◯ LOW | IMPORTANT |

**CI:** Confidence interval

1. Only direct comparisons from the Network Meta-analysis by Leucht et al. (2013) were considered.
2. Details for each of the included studies are limited. According to the overall quality of included studies (appendix 6b), details of the assessment masking are lacking in about half of the studies and the risk of attrition bias is high in almost 50% of the studies. However, we cannot rely on the assumption that such trend can be simply extended to the studies included in this specific head-to-head comparison. Thus, we applied a downgrade of -1 instead of -2.
3. Details not available for this particular comparison.
4. None of the included trials was performed in a low-resources humanitarian setting. Further, data are from the general population of people suffering from psychosis rather than people not responding to a first-line FGA, as this was the best available evidence, although fairly approximate.
5. The analysis includes 792 patients. The 95% CrI in relatively narrow.

**Author(s)**: Giovanni Ostuzzi

**Date**: 27/01/2016

**Question**: Aripiprazole compared to haloperidol for people suffering from psychosis (including schizophrenia) who failed to improve after treatment with one FGA

**Setting**: humanitarian/low-resources setting

**Bibliography**: Leucht S, Cipriani A, Spineli L, Mavridis D, Orey D, Richter F, Samara M, Barbui C, Engel RR, Geddes JR, Kissling W, Stapf MP, Lässig B, Salanti G, Davis JM. Comparative efficacy and tolerability of 15 antipsychotic medications in schizophrenia: a multiple-treatments meta-analysis. Lancet 2013, 14;382(9896):951-62.

| **Quality assessment** | | | | | | | **№ of patients** | | **Effect** | | **Quality** | **Importance** |
| --- | --- | --- | --- | --- | --- | --- | --- | --- | --- | --- | --- | --- |
| **№ of studies** | **Study design** | **Risk of bias** | **Inconsistency** | **Indirectness** | **Imprecision** | **Other considerations** | **aripiprazole** | **haloperidol** | **Relative (95% CI)** | **Absolute (95% CI)** |  |  |
| Efficacy (follow up: range 4 weeks to 12 weeks; assessed with: PANSS or BPRS total score) | | | | | | | | | | | | |
| 4 | randomised trials ^1^ | serious ^2^ | not serious ^3^ | serious ^4^ | not serious ^5^ | none | 1806 patients included in the analysis. Not clear how many patients were allocated to each arm. The SMD is on the edge of significance in favour of aripiprazole: SMD 0.02, 95% CrI (Credibility Interval) -0.08 to 0.12. | | | | ⨁⨁◯◯ LOW | CRITICAL |
| Acceptability (follow up: range 4 weeks to 12 weeks; assessed with: n. of patients leaving the study early for any reason) | | | | | | | | | | | | |
| 4 | randomised trials ^1^ | serious ^2^ | not serious ^3^ | serious ^4^ | not serious ^5^ | none | 1806 patients included in the analysis. Not clear how many patients were allocated to each arm. The OR significantly favours aripiprazole: OR 1.36, 95% CrI (Credibility Interval) 1.11 to 1.67. | | | | ⨁⨁◯◯ LOW | CRITICAL |
| Weight gain (follow up: range 4 weeks to 12 weeks; assessed with: weight gain at the study endpoint) | | | | | | | | | | | | |
| 4 | randomised trials ^1^ | serious ^2^ | not serious ^3^ | serious ^4^ | not serious ^5^ | none | 1806 patients included in the analysis. Not clear how many patients were allocated to each arm. The SMD does not show the superiority of one intervention over the other: SMD -0.03, 95% CrI (Credibility Interval) -0.27 to 0.21 (negative SMDs favour haloperidol). | | | | ⨁⨁◯◯ LOW | IMPORTANT |
| Prolactin (follow up: range 4 weeks to 12 weeks; assessed with: serum concentration at study endpoint) | | | | | | | | | | | | |
| 4 | randomised trials ^1^ | serious ^2^ | not serious ^3^ | serious ^4^ | not serious ^5^ | none | 1806 patients included in the analysis. Not clear how many patients were allocated to each arm. The SMD significantly favours aripiprazole: SMD 0.85, 95% CrI (Credibility Interval) 0.53 to 1.18. | | | | ⨁⨁◯◯ LOW | IMPORTANT |
| Motor symptoms (follow up: range 4 weeks to 12 weeks; assessed with: use of antiparkinson medications) | | | | | | | | | | | | |
| 4 | randomised trials ^1^ | serious ^2^ | not serious ^3^ | serious ^4^ | not serious ^5^ | none | 1806 patients included in the analysis. Not clear how many patients were allocated to each arm. The OR significantly favours aripiprazole: OR 2.96, 95% CrI (Credibility Interval) 1.63 to 5.36. | | | | ⨁⨁◯◯ LOW | IMPORTANT |
| Sedation (follow up: range 4 weeks to 12 weeks; assessed with: n. of patients experiencing sedation) | | | | | | | | | | | | |
| 4 | randomised trials ^1^ | serious ^2^ | not serious ^3^ | serious ^4^ | not serious ^5^ | none | 1806 patients included in the analysis. Not clear how many patients were allocated to each arm. The OR significantly favours aripiprazole: OR 1.80, 95% CrI (Credibility Interval) 0.82 to 3.94. | | | | ⨁⨁◯◯ LOW | IMPORTANT |

**CI:** Confidence interval

1. Only direct comparisons from the Network Meta-analysis by Leucht et al. (2013) were considered.
2. Details for each of the included studies are limited. According to the overall quality of included studies (appendix 6b), details of the assessment masking are lacking in about half of the studies and the risk of attrition bias is high in almost 50% of the studies. However, we cannot rely on the assumption that such trend can be simply extended to the studies included in this specific head-to-head comparison. Thus, we applied a downgrade of -1 instead of -2.
3. Details not available for this particular comparison.
4. None of the included trials was performed in a low-resources humanitarian setting. Further, data are from the general population of people suffering from psychosis rather than people not responding to a first-line FGA, as this was the best available evidence, although fairly approximate.
5. 1806 patients were included in the analysis. The 95% CrI is relatively narrow.

**Author(s)**: Giovanni Ostuzzi

**Date**: 27/01/2016

**Question**: Olanzapine compared to risperidone for people suffering from psychosis (including schizophrenia) who failed to improve after treatment with one FGA

**Setting**: Humanitarian/low-resources setting

**Bibliography**: Komossa K, Rummel-Kluge C, Schwarz S, Schmid F, Hunger H, Kissling W, Leucht S. Risperidone versus other atypical antipsychotics for schizophrenia. Cochrane Database of Systematic Reviews 2011, Issue 1. Art. No.: CD006626.

| **Quality assessment** | | | | | | | **№ of patients** | | **Effect** | | **Quality** | **Importance** |
| --- | --- | --- | --- | --- | --- | --- | --- | --- | --- | --- | --- | --- |
| **№ of studies** | **Study design** | **Risk of bias** | **Inconsistency** | **Indirectness** | **Imprecision** | **Other considerations** | **olanzapine** | **risperidone** | **Relative (95% CI)** | **Absolute (95% CI)** |  |  |
| Treatment response (assessed with: n. of patients with no significant response) | | | | | | | | | | | | |
| 7 | randomised trials | very serious ^1^ | not serious ^2^ | serious ^3^ | not serious ^4^ | none | 397/691 (57.5%) | 406/685 (59.3%) | **RR 1.06** (0.99 to 1.13) | **36 more per 1000** (from 6 fewer to 77 more) | ⨁◯◯◯ VERY LOW | CRITICAL |
| Symptoms severity (long-term) (follow up: 26 or more weeks; assessed with: BPRS average endpoint score. Lower scores indicate less severe psychopathology; negative values of the MD favour risperidone.; Scale from: 18 to 126) | | | | | | | | | | | | |
| 2 | randomised trials | very serious ^5^ | serious ^6^ | serious ^3^ | not serious ^7^ | none | 198 | 195 | - | MD **4.28 higher** (1.34 lower to 9.91 higher) | ⨁◯◯◯ VERY LOW | CRITICAL |
| Quality of life (assessed with: QLS total score. Higher scores indicate lower quality of life; negative values of the MD favour risperidone) | | | | | | | | | | | | |
| 2 | randomised trials | very serious ^5^ | not serious ^2^ | serious ^3^ | not serious ^8^ | none | 148 | 148 | - | MD **5.1 higher** (1.09 higher to 9.1 higher) | ⨁◯◯◯ VERY LOW | IMPORTANT |
| Acceptability (assessed with: n. of patients leaving the study early for any reason) | | | | | | | | | | | | |
| 16 | randomised trials | very serious ^9^ | not serious ^2^ | serious ^3^ | not serious ^10^ | none | 653/1365 (47.8%) | 762/1373 (55.5%) | **RR 1.14** (1.07 to 1.21) | **78 more per 1000** (from 39 more to 117 more) | ⨁◯◯◯ VERY LOW | CRITICAL |
| Tolerability (assessed with: n. of patients leaving the study early due to adverse events) | | | | | | | | | | | | |
| 13 | randomised trials | very serious ^11^ | not serious ^12^ | serious ^3^ | not serious ^13^ | none | 153/1294 (11.8%) | 138/1301 (10.6%) | **RR 0.96** (0.71 to 1.30) | **4 fewer per 1000** (from 31 fewer to 32 more) | ⨁◯◯◯ VERY LOW | CRITICAL |
| Sedation (assessed with: n. of patients experiencing sedation) | | | | | | | | | | | | |
| 11 | randomised trials | very serious ^14^ | not serious ^15^ | serious ^3^ | not serious ^16^ | none | 411/1289 (31.9%) | 388/1287 (30.1%) | **RR 0.93** (0.84 to 1.04) | **21 fewer per 1000** (from 12 more to 48 fewer) | ⨁◯◯◯ VERY LOW | IMPORTANT |
| Weight gain (assessed with: n. of patients experiencing significant weight gain) | | | | | | | | | | | | |
| 8 | randomised trials | very serious ^17^ | serious ^18^ | serious ^3^ | not serious ^19^ | none | 302/938 (32.2%) | 168/935 (18.0%) | **RR 0.54** (0.39 to 0.76) | **83 fewer per 1000** (from 43 fewer to 110 fewer) | ⨁◯◯◯ VERY LOW | IMPORTANT |
| Motor symptoms (assessed with: n. of patients taking antiparkinson medications) | | | | | | | | | | | | |
| 13 | randomised trials | serious ^20^ | not serious ^21^ | serious ^3^ | not serious ^22^ | none | 241/1296 (18.6%) | 312/1303 (23.9%) | **RR 1.28** (1.06 to 1.55) | **67 more per 1000** (from 14 more to 132 more) | ⨁⨁◯◯ LOW | IMPORTANT |
| Prolactin (assessed with: plasma concentration change from baseline in ng/ml; positive values of the MD favour olanzapine) | | | | | | | | | | | | |
| 6 | randomised trials | very serious ^23^ | serious ^18^ | serious ^3^ | not serious ^24^ | none | 649 | 642 | - | MD **22.84 higher** (17.69 higher to 27.98 higher) | ⨁◯◯◯ VERY LOW | IMPORTANT |

**CI:** Confidence interval; **RR:** Risk ratio; **MD:** Mean difference

1. For most studies it is not clear whether the assessment was masked or not, however this may be a problem of reporting rather than a true detection bias. The dropout rate is higher than 30% for 4 of the 7 studies included.
2. I-squared=0%
3. None of the included trials was performed in low-resources settings. Further, data are from the general population of people suffering from psychosis rather than people not responding to a first-line FGA, as this was the best available evidence, although fairly approximate.
4. The overall number of individuals enrolled is 1376. The 95% CI is narrow.
5. For most studies it is not clear whether the assessment was masked or not, however this may be a problem of reporting rather than a true detection bias. The dropout rate is higher than 30% for both the included studies.
6. I-squared=69%
7. The overall number of individuals enrolled is 392. The 95% CI is relatively narrow.
8. The overall number of individuals enrolled is 296. The 95% CI is relatively narrow.
9. For most studies it is not clear whether the assessment was masked or not, however this may be a problem of reporting rather than a true detection bias. The dropout rate is higher than 30% for 11 of the 16 studies included.
10. The overall number of individuals enrolled is 2738. The 95% CI is narrow.
11. For most studies it is not clear whether the assessment was masked or not, however this may be a problem of reporting rather than a true detection bias. The dropout rate is higher than 30% for 10 of the 13 included studies.
12. I-squared=31%
13. The overall number of individuals enrolled is 1595. The 95% CI is narrow.
14. For most studies it is not clear whether the assessment was masked or not, however this may be a problem of reporting rather than a true detection bias. The dropout rate is higher than 30% for 8 of the 11 included studies.
15. I-squared=4%
16. The overall number of individuals enrolled is 2576. The 95% CI is narrow.
17. For most studies it is not clear whether the assessment was masked or not, however this may be a problem of reporting rather than a true detection bias. The dropout rate is higher than 30% for 5 of the 8 included studies.
18. I-squared=65%
19. The overall number of individuals enrolled is 1873. The 95% CI is narrow.
20. For most studies it is not clear whether the assessment was masked or not, however this may be a problem of reporting rather than a true detection bias. The dropout rate is higher than 30% for of 8 the 13 included studies.
21. I-squared=28%
22. The overall number of individuals enrolled is 2599. The 95% CI is narrow.
23. For most studies it is not clear whether the assessment was masked or not, however this may be a problem of reporting rather than a true detection bias. The dropout rate is higher than 30% for of 4 the 6 included studies.
24. The overall number of individuals enrolled is 1291. The 95% CI is relatively narrow.

**(3) Which individual antipsychotic is effective and safe in people with a diagnosis of treatment-resistant psychosis?**

**Population/Intervention(s)/Comparator/Outcome(s) (PICO)**

- Population: people suffering from treatment-resistant psychosis (including schizophrenia)
- Intervention: clozapine/risperidone/olanzapine/FGAs
- Comparison: clozapine/risperidone/olanzapine/FGAs
- Treatment response
- Remission
- Symptoms severity
- Functioning
- Quality of life
- Acceptability
- Tolerability
- Adverse events: endocrine (prolactin increase), motor symptoms, weight gain, sedation, blood problems
- Setting: humanitarian, low-resources settings

**List of the systematic reviews identified by the search process**

*INCLUDED IN GRADE TABLES OR FOOTNOTES*

Asenjo Lobos C, Komossa K, Rummel-Kluge C, Hunger H, Schmid F, Schwarz S, Leucht S. Clozapine versus other atypical antipsychotics for schizophrenia. Cochrane Database of Systematic Reviews 2010, Issue 11. Art. No.: CD006633.

Duggan L, Fenton M, Rathbone J, Dardennes R, El-Dosoky A, Indran S. Olanzapine for schizophrenia. Cochrane Database of Systematic Reviews 2005, Issue 2. Art. No.: CD001359.

Essali A, Al-Haj Haasan N, Li C, Rathbone J. Clozapine versus typical neuroleptic medication for schizophrenia. Cochrane Database of Systematic Reviews 2009, Issue 1. Art. No.: CD000059.

Samara MT, Dold M, Gianatsi M, Nikolakopoulou A, Helfer B, Salanti G, Leucht S. Efficacy, Acceptability, and Tolerability of Antipsychotics in Treatment-Resistant Schizophrenia. A Network Meta-analysis. JAMA Psychiatry. Published online February 3, 2016. doi:10.1001/jamapsychiatry.2015.2955

**PICO table**

| **Serial n.** | **Intervention/comparison** | **Outcomes** | **Systematic reviews used for GRADE** | **Explanation** |
| --- | --- | --- | --- | --- |
| **I** | clozapine/FGAs | Treatment response  Remission  Symptoms severity  Functioning  Quality of life  Acceptability  Tolerability  Adverse events:   - endocrine (prolactin) - motor symptoms - weight gain - sedation - blood problems | Essali et al., 2009  Essali et al., 2009  Essali et al., 2009  Data not available  Data not available  Essali et al., 2009  Data not available  Data not available  Essali et al., 2009  Essali et al., 2009  Data not available  Essali et al., 2009 |  |
| **II** | clozapine/risperidone | Treatment response  Remission  Symptoms severity  Functioning  Quality of life  Acceptability  Tolerability  Adverse events:   - endocrine (prolactin) - motor symptoms - weight gain - sedation - blood problems | Samara et al., 2016  Data not available  Samara et al., 2016  Data not available  Data not available  Samara et al., 2016  Data not available  Data not available  Data not available  Data not available  Data not available  Data not available |  |
| **III** | clozapine/olanzapine | Treatment response  Remission  Symptoms severity  Functioning  Quality of life  Acceptability  Tolerability  Adverse events:   - endocrine (prolactin) - motor symptoms - weight gain - sedation - blood problems | Samara et al., 2016  Data not available  Samara et al., 2016  Data not available  Data not available  Samara et al., 2016  Data not available  Data not available  Data not available  Data not available  Data not available  Data not available |  |
| **IV** | olanzapine/FGAs | Treatment response  Remission  Symptoms severity  Functioning  Quality of life  Acceptability  Tolerability  Adverse events:   - endocrine (prolactin) - motor symptoms - weight gain - sedation - blood problems | Duggan et al., 2005  Data not available  Data not available  Data not available  Data not available  Duggan et al., 2005  Data not available  Data not available  Data not available  Data not available  Data not available  Data not available |  |
| **V** | olanzapine/SGAs | Treatment response  Remission  Symptoms severity  Functioning  Quality of life  Acceptability  Tolerability  Adverse events:   - endocrine (prolactin) - motor symptoms - weight gain - sedation | Duggan et al., 2005  Data not available  Duggan et al., 2005  Data not available  Data not available  Duggan et al., 2005  Data not available  Data not available  Duggan et al., 2005  Duggan et al., 2005  Duggan et al., 2005 |  |

**GRADE tables**

**Author(s)**: Giovanni Ostuzzi

**Date**: 27/01/2016

**Question**: Clozapine compared to FGAs for people with treatment-resistant psychosis (including schizophrenia)

**Setting**: humanitarian/low-resources setting

**Bibliography**: Essali A, Al-Haj Haasan N, Li C, Rathbone J. Clozapine versus typical neuroleptic medication for schizophrenia. Cochrane Database of Systematic Reviews 2009, Issue 1. Art. No.: CD000059.

| **Quality assessment** | | | | | | | **№ of patients** | | **Effect** | | **Quality** | **Importance** |
| --- | --- | --- | --- | --- | --- | --- | --- | --- | --- | --- | --- | --- |
| **№ of studies** | **Study design** | **Risk of bias** | **Inconsistency** | **Indirectness** | **Imprecision** | **Other considerations** | **clozapine** | **FGAs** | **Relative (95% CI)** | **Absolute (95% CI)** |  |  |
| Efficacy (short term) (assessed with: relapse rate) | | | | | | | | | | | | |
| 4 | randomised trials | not serious ^1^ | not serious ^2^ | serious ^3^ | not serious ^4^ | none | 23/190 (12.1%) | 24/206 (11.7%) | **RR 1.04** (0.61 to 1.78) | **5 more per 1000** (from 45 fewer to 91 more) | ⨁⨁⨁◯ MODERATE | CRITICAL |
| Remission (short term) (assessed with: n. of patients not clinically improved) | | | | | | | | | | | | |
| 4 | randomised trials | not serious ^5^ | not serious ^2^ | serious ^3^ | not serious ^6^ | none | 118/178 (66.3%) | 179/192 (93.2%) | **RR 0.71** (0.64 to 0.79) | **270 fewer per 1000** (from 196 fewer to 336 fewer) | ⨁⨁⨁◯ MODERATE | CRITICAL |
| Symptoms severity (assessed with: BPRS endpoint score. Lower scores less severe psychopathology; negative values of the MD favour clozapine; Scale from: 18 to 126) | | | | | | | | | | | | |
| 5 | randomised trials | not serious ^7^ | serious ^8^ | serious ^3^ | not serious ^9^ | none | 208 | 221 | - | MD **7.83 lower** (10.01 lower to 5.64 lower) | ⨁⨁◯◯ LOW | IMPORTANT |
| Acceptability (short term) (assessed with: n. of patients leaving the study early) | | | | | | | | | | | | |
| 5 | randomised trials | not serious ^7^ | not serious ^2^ | serious ^3^ | not serious ^9^ | none | 30/211 (14.2%) | 27/225 (12.0%) | **RR 1.19** (0.73 to 1.94) | **23 more per 1000** (from 32 fewer to 113 more) | ⨁⨁⨁◯ MODERATE | CRITICAL |
| Blood problems (assessed with: n. of patients with blood problems) | | | | | | | | | | | | |
| 5 | randomised trials | not serious ^10^ | not serious ^2^ | serious ^3^ | serious ^11^ | none | 21/400 (5.3%) | 11/427 (2.6%) | **RR 1.90** (0.97 to 3.71) | **23 more per 1000** (from 1 fewer to 70 more) | ⨁⨁◯◯ LOW | IMPORTANT |
| Weight gain (assessed with: n. of patients experiencing weight gain) | | | | | | | | | | | | |
| 3 | randomised trials | very serious ^12^ | serious ^13^ | serious ^3^ | not serious ^14^ | none | 135/236 (57.2%) | 107/248 (43.1%) | **RR 1.33** (1.11 to 1.59) | **142 more per 1000** (from 47 more to 255 more) | ⨁◯◯◯ VERY LOW | IMPORTANT |
| Motor symptoms (assessed with: n. of patients experiencing movement disorders) | | | | | | | | | | | | |
| 4 | randomised trials | serious ^15^ | not serious ^16^ | serious ^3^ | not serious ^17^ | none | 120/257 (46.7%) | 161/264 (61.0%) | **RR 0.77** (0.67 to 0.90) | **140 fewer per 1000** (from 61 fewer to 201 fewer) | ⨁⨁◯◯ LOW | IMPORTANT |

**CI:** Confidence interval; **RR:** Risk ratio; **MD:** Mean difference

1. The attrition rate was <30% for each of the 4 included studies. The masking of assessment was not clearly reported, however this may be a problem of reporting rather than a true detection bias.
2. I-squared=0%
3. None of the included trials was performed in a low-resources humanitarian setting.
4. 396 individuals were included in the analysis. The 95% CI was relatively narrow.
5. None of the included studies had an attrition rate higher than 30%.
6. 370 individuals were included in the analysis. The 95% CI show an advantage for clozapine and was relatively narrow.
7. The attrition rate was <30% for each of the 5 included studies. The masking of assessment was not clearly reported, however this may be a problem of reporting rather than a true detection bias.
8. I-squared=65%
9. Over 400 individuals were included in the analysis. The 95% CI was relatively narrow.
10. The attrition rate was <30% for four of the five included studies. The masking of assessment was not clearly reported, however this may be a problem of reporting rather than a true detection bias.
11. 827 individuals were included in the analysis. The 95% CI crosses both 1 (no effect) and 2 (appreciable benefit).
12. The attrition rate was very high (about 58%) for one of the three included studies. For none of them the masking of assessment was clearly reported.
13. I-squared=61%
14. 827 individuals were included in the analysis. The 95% CI is relatively narrow.
15. The attrition rate was <30% for three of the four included studies. The study which gives the largest contribution to the analysis had a very high attrition rate (about 58%). For none of them the masking of assessment was clearly reported.
16. I-squared=35%
17. 521 individuals were included in the analysis. The 95% CI is relatively narrow.

**Author(s)**: Giovanni Ostuzzi

**Date**: 04/02/2016

**Question**: Clozapine compared to risperidone for people with treatment-resistant psychosis (including schizophrenia)

**Setting**: humanitarian/low-resources setting

**Bibliography**: Samara MT, Dold M, Gianatsi M, Nikolakopoulou A, Helfer B, Salanti G, Leucht S. Efficacy, Acceptability, and Tolerability of Antipsychotics in Treatment-Resistant Schizophrenia. A Network Meta-analysis. JAMA Psychiatry. Published online February 3, 2016. doi:10.1001/jamapsychiatry.2015.2955

| **Quality assessment** | | | | | | | **№ of patients** | | **Effect** | | **Quality** | **Importance** |
| --- | --- | --- | --- | --- | --- | --- | --- | --- | --- | --- | --- | --- |
| **№ of studies** | **Study design** | **Risk of bias** | **Inconsistency** | **Indirectness** | **Imprecision** | **Other considerations** | **clozapine** | **risperidone** | **Relative (95% CI)** | **Absolute (95% CI)** |  |  |
| Symptoms severity (follow up: mean 12 weeks; assessed with: mean score reduction on BPRS, PANNS or other validated scales) | | | | | | | | | | | | |
| 6 | randomised trials | not serious ^1^ | not serious ^2^ | serious ^3^ | not serious ^4^ | none | 596 patients included in the analysis. Not clear how many patients were allocated to each arm. The SMD does not show any advantage of one treatment over the other: SMD -0.04, 95% CrI (Credibility Interval) -0.25 to 0.18 (negative values favour clozapine). | | | | ⨁⨁⨁◯ MODERATE | CRITICAL |
| Efficacy (treatment response) (follow up: mean 12 weeks; assessed with: at least a 20% reduction of PANSS or BPRS or at least minimal improvement on the CGI) | | | | | | | | | | | | |
| 4 | randomised trials | not serious ^5^ | not serious ^2^ | serious ^3^ | not serious ^6^ | none | 408 patients included in the analysis. Not clear how many patients were allocated to each arm. The OR does not show any advantage of one treatment over the other: OR 0.98, 95% CrI (Credibility Interval) 0.55 to 1.75 (OR>1 favour clozapine). | | | | ⨁⨁⨁◯ MODERATE | CRITICAL |
| Acceptability (follow up: mean 12 weeks; assessed with: attrition due to any cause) | | | | | | | | | | | | |
| 6 | randomised trials | not serious ^1^ | not serious ^2^ | serious ^3^ | not serious ^7^ | none | 587 patients included in the analysis. Not clear how many patients were allocated to each arm. The OR does not show any advantage of one treatment over the other: OR 0.97, 95% CrI (Credibility Interval) 0.54 to 1.72 (OR>1 favour clozapine). | | | | ⨁⨁⨁◯ MODERATE | CRITICAL |

**CI:** Confidence interval

1. Details for each of the included studies are limited. According to eAppendix 4, eFigure 1 and 2, details of the risk of detection bias was low for all the included studies and the risk of attrition bias was high for 2 studies over 7 including such comparison, unclear for 4 studies and low for one study. However, note that only 6 over 7 studies were included in this analysis, and this quality interpretation is therefor rather approximate.
2. Details not available for this particular comparison.
3. None of the included trials was performed in low-resources settings.
4. 596 patients were included in the analysis. The 95% CrI is relatively narrow.
5. Details for each of the included studies are limited. According to eAppendix 4, eFigure 1 and 2, details of the risk of detection bias was low for all the included studies and the risk of attrition bias was high for 2 studies over 7 including such comparison, unclear for 4 studies and low for one study. However, note that only 4 over 7 studies were included in this analysis, and this quality interpretation is therefor rather approximate.
6. 408 patients were included in the analysis. The 95% CrI is relatively narrow.
7. 587 patients were included in the analysis. The 95% CrI is relatively narrow.

**Author(s)**: Giovanni Ostuzzi

**Date**: 04/02/2016

**Question**: Clozapine compared to olanzapine for people with treatment-resistant psychosis (including schizophrenia)

**Setting**: humanitarian/low-resources settings

**Bibliography**: Samara MT, Dold M, Gianatsi M, Nikolakopoulou A, Helfer B, Salanti G, Leucht S. Efficacy, Acceptability, and Tolerability of Antipsychotics in Treatment-Resistant Schizophrenia. A Network Meta-analysis. JAMA Psychiatry. Published online February 3, 2016. doi:10.1001/jamapsychiatry.2015.2955

| **Quality assessment** | | | | | | | **№ of patients** | | **Effect** | | **Quality** | **Importance** |
| --- | --- | --- | --- | --- | --- | --- | --- | --- | --- | --- | --- | --- |
| **№ of studies** | **Study design** | **Risk of bias** | **Inconsistency** | **Indirectness** | **Imprecision** | **Other considerations** | **clozapine** | **olanzapine** | **Relative (95% CI)** | **Absolute (95% CI)** |  |  |
| Symptoms severity (follow up: mean 17 weeks; assessed with: mean score reduction on BPRS, PANNS or other validated scales) | | | | | | | | | | | | |
| 7 | randomised trials | not serious ^1^ | not serious ^2^ | serious ^3^ | not serious ^4^ | none | 596 patients included in the analysis. Not clear how many patients were allocated to each arm. The SMD does not show any advantage of one treatment over the other: SMD -0.09, 95% CrI (Credibility Interval) -0.28 to 0.12 (negative values favour olanzapine). | | | | ⨁⨁⨁◯ MODERATE | CRITICAL |
| Efficacy (treatment response) (follow up: mean 17 weeks; assessed with: at least a 20% reduction of PANSS or BPRS or at least minimal improvement on the CGI) | | | | | | | | | | | | |
| 5 | randomised trials | not serious ^5^ | not serious ^2^ | serious ^3^ | not serious ^6^ | none | 494 patients included in the analysis. Not clear how many patients were allocated to each arm. The OR does not show any advantage of one treatment over the other: OR 1.04, 95% CrI (Credibility Interval) 0.63 to 1.79 (OR>1 favour clozapine). | | | | ⨁⨁⨁◯ MODERATE | CRITICAL |
| Acceptability (follow up: mean 17 weeks; assessed with: attrition due to any cause) | | | | | | | | | | | | |
| 7 | randomised trials | not serious ^1^ | not serious ^2^ | serious ^3^ | serious ^7^ | none | 596 patients included in the analysis. Not clear how many patients were allocated to each arm. The OR does not show any advantage of one treatment over the other: OR 0.78, 95% CrI (Credibility Interval) 0.45 to 1.32 (OR>1 favour olanzapine). | | | | ⨁⨁◯◯ LOW | CRITICAL |

**CI:** Confidence interval

1. Details for each of the included studies are limited. According to eAppendix 4, eFigure 1 and 2, details of the risk of detection bias was low for all the included studies and the risk of attrition bias was high for 1 and unclear for 1 over 7 studies.
2. Details not available for this particular comparison.
3. None of the included trials was performed in low-resources settings.
4. 596 patients were included in the analysis. The 95% CrI is relatively narrow.
5. Details for each of the included studies are limited. According to eAppendix 4, eFigure 1 and 2, details of the risk of detection bias was low for all the included studies and the risk of attrition bias was high for 1 and unclear for 1 over 7 studies. However, note that only 5 over 7 studies were included in this analysis, and this quality interpretation is therefor rather approximate.
6. 494 patients were included in the analysis. The 95% CrI is relatively narrow.
7. 596 patients were included in the analysis. The 95% CrI crosses both 1 (no effect) and 0.5 (appreciable harm).

**Author(s)**: Giovanni Ostuzzi

**Date**: 27/01/2016

**Question**: Olanzapine compared to FGAs for people with treatment-resistant psychosis (including schizophrenia)

**Setting**: humanitarian/low-resources setting

**Bibliography**: Duggan L, Fenton M, Rathbone J, Dardennes R, El-Dosoky A, Indran S. Olanzapine for schizophrenia. Cochrane Database of Systematic Reviews 2005, Issue 2. Art. No.: CD001359.

| **Quality assessment** | | | | | | | **№ of patients** | | **Effect** | | **Quality** | **Importance** |
| --- | --- | --- | --- | --- | --- | --- | --- | --- | --- | --- | --- | --- |
| **№ of studies** | **Study design** | **Risk of bias** | **Inconsistency** | **Indirectness** | **Imprecision** | **Other considerations** | **olanzapine** | **FGAs** | **Relative (95% CI)** | **Absolute (95% CI)** |  |  |
| Efficacy (follow up: range 2 weeks to 8 weeks; assessed with: no relevant clinical response) | | | | | | | | | | | | |
| 1 | randomised trials | not serious ^1^ | not serious | serious ^2^ | serious ^3^ | none | 39/42 (92.9%) | 42/42 (100.0%) | **RR 0.93** (0.85 to 1.02) | **70 fewer per 1000** (from 20 more to 150 fewer) | ⨁⨁◯◯ LOW | CRITICAL |
| Acceptability (follow up: range 2 weeks to 8 weeks; assessed with: n. of patients leaving the study early for any reason) | | | | | | | | | | | | |
| 1 | randomised trials | not serious ^1^ | not serious | serious ^2^ | very serious ^4^ | none | 12/42 (28.6%) | 13/42 (31.0%) | **RR 0.92** (0.48 to 1.78) | **25 fewer per 1000** (from 161 fewer to 241 more) | ⨁◯◯◯ VERY LOW | CRITICAL |

**CI:** Confidence interval; **RR:** Risk ratio

1. The attrition rate was low (8%). The masking of assessment was not clearly reported, however this may be a problem of reporting rather than a true detection bias.
2. The included study was not performed in a low-resources setting.
3. The study included 84 individuals. The 95% CI is narrow and shows no differences between treatments.
4. The study included 84 individuals. The 95% CI includes 1 (no effect) and 0.5 (appreciable harm).

**Author(s)**: Giovanni Ostuzzi

**Date**: 27/01/2016

**Question**: Olanzapine compared to SGAs for people with treatment-resistant psychosis (including schizophrenia)

**Setting**: humanitarian/low-resources setting

**Bibliography**: Duggan L, Fenton M, Rathbone J, Dardennes R, El-Dosoky A, Indran S. Olanzapine for schizophrenia. Cochrane Database of Systematic Reviews 2005, Issue 2. Art. No.: CD001359.

| **Quality assessment** | | | | | | | **№ of patients** | | **Effect** | | **Quality** | **Importance** |
| --- | --- | --- | --- | --- | --- | --- | --- | --- | --- | --- | --- | --- |
| **№ of studies** | **Study design** | **Risk of bias** | **Inconsistency** | **Indirectness** | **Imprecision** | **Other considerations** | **olanzapine** | **SGAs** | **Relative (95% CI)** | **Absolute (95% CI)** |  |  |
| Efficacy (follow up: 18 weeks; assessed with: n. of patients without a clinically relevant response measured as an improvement lower than 50% at CGI) | | | | | | | | | | | | |
| 2 | randomised trials | very serious ^1^ | not serious ^2^ | serious ^3^ | not serious ^4^ | none | 86/166 (51.8%) | 96/164 (58.5%) | **RR 0.89** (0.73 to 1.08) | **64 fewer per 1000** (from 47 more to 158 fewer) | ⨁◯◯◯ VERY LOW | CRITICAL |
| Symptoms severity (follow up: 18 weeks; assessed with: PANNS endpoint score. Higher scores indicate more severe psychopathology; negative values of the MD favour olanzapine; Scale from: 30 to 210) | | | | | | | | | | | | |
| 1 | randomised trials | very serious ^5^ | not serious | serious ^3^ | not serious ^6^ | none | 89 | 87 | - | MD **0.03 higher** (8.07 lower to 8.13 higher) | ⨁◯◯◯ VERY LOW | CRITICAL |
| Acceptability (follow up: range 8 weeks to 24 weeks; assessed with: n. of patients leaving the study early due to any reason) | | | | | | | | | | | | |
| 4 | randomised trials | very serious ^7^ | not serious ^2^ | serious ^3^ | not serious ^8^ | none | 105/231 (45.5%) | 105/226 (46.5%) | **RR 0.98** (0.81 to 1.19) | **9 fewer per 1000** (from 88 fewer to 88 more) | ⨁◯◯◯ VERY LOW | CRITICAL |
| Motor symptoms (follow up: 18 weeks; assessed with: SAS scale. Higher scores indicate higher adverse events; negative values of the MD favour olanzapine) | | | | | | | | | | | | |
| 1 | randomised trials | very serious ^5^ | not serious | serious ^3^ | not serious ^9^ | none | 69 | 70 | - | MD **0.1 lower** (1.56 lower to 1.36 higher) | ⨁◯◯◯ VERY LOW | IMPORTANT |
| Sedation (assessed with: n. of patients experiencing sedation) | | | | | | | | | | | | |
| 2 | randomised trials | very serious ^1^ | not serious ^2^ | serious ^3^ | not serious ^10^ | none | 20/166 (12.0%) | 37/164 (22.6%) | **RR 0.54** (0.32 to 0.88) | **104 fewer per 1000** (from 27 fewer to 153 fewer) | ⨁◯◯◯ VERY LOW | IMPORTANT |
| Weight gain (assessed with: n. of patients experiencing weight gain) | | | | | | | | | | | | |
| 2 | randomised trials | very serious ^1^ | not serious ^2^ | serious ^3^ | serious ^11^ | none | 19/166 (11.4%) | 17/164 (10.4%) | **RR 1.10** (0.60 to 2.03) | **10 more per 1000** (from 41 fewer to 107 more) | ⨁◯◯◯ VERY LOW | IMPORTANT |

**CI:** Confidence interval; **RR:** Risk ratio; **MD:** Mean difference

1. Both the included studies had an attrition rate higher than 30%.
2. I-squared=0%
3. None of the included trials was performed in a low-resources humanitarian setting.
4. The overall number of individuals included was 330. The 95% CI shows no difference between treatments and is relatively narrow.
5. The attrition rate of the included study was higher than 30%.
6. The overall number of individuals included was 176. The 95% CI shows no difference between treatments and is relatively narrow.
7. The masking of assessment was not clearly reported, however this may be a problem of reporting rather than a true detection bias. For 3 of the 4 included studies the attrition rate was higher than 30%.
8. The overall number of individuals included was 457. The 95% CI shows no difference between treatments and is relatively narrow.
9. The overall number of individuals included was 139. The 95% CI shows no difference between treatments and is relatively narrow.
10. The overall number of individuals included was 330. The 95% CI shows a significant advantage for olanzapine.
11. The overall number of individuals included was 330. The 95% CI shows includes 1 (no effect) and 2 (appreciable benefit).

**(4) Are antidepressants - antipsychotics combinations effective and safe in people with psychosis-related depressive, cognitive and negative symptoms?**

**Population/Intervention(s)/Comparator/Outcome(s) (PICO)**

- Population: people suffering from psychosis-related depressive, cognitive and negative symptoms
- Intervention: antidepressants plus antipsychotics
- Comparison: antipsychotics alone or antipsychotics plus placebo
- Outcomes:
- Treatment response
- Remission
- Symptoms severity
- Functioning
- Quality of life
- Acceptability
- Tolerability
- Adverse events: endocrine (prolactin increase), motor symptoms, weight gain, sedation, blood problems
- Setting: humanitarian, low-resources settings

**List of the systematic reviews identified by the search process**

*INCLUDED IN GRADE TABLES OR FOOTNOTES*

Helfer B, Samara MT, Huhn M, Klupp E, Leucht C, Zhu Y, Engel RR, Leucht S. Efficacy and Safety of Antidepressants Added to Antipsychotics for Schizophrenia: A Systematic Review and Meta-Analysis. Am J Psychiatry. 2016 Jun 10:appiajp201615081035

Singh SP, Singh V, Kar N, Chan K. Efficacy of antidepressants in treating the negative symptoms of chronic schizophrenia: meta-analysis. Br J Psychiatry. 2010;197(3):174-9.

Rummel-Kluge C, Kissling W, Leucht S . Antidepressants for the negative symptoms of schizophrenia. Cochrane Database of Systematic Reviews 2006, Issue 3. Art. No.: CD005581.

Vernon JA, Grudnikoff E, Seidman AJ, Frazier TW, Vemulapalli MS, Pareek P, Goldberg TE, Kane JM, Correll CU. Antidepressants for cognitive impairment in schizophrenia - a systematic review and meta-analysis. Schizophr Res 2014;159(2-3):385-94

**PICO table**

| **Serial n.** | **Intervention/comparison** | **Outcomes** | **Systematic reviews used for GRADE** | **Explanation** |
| --- | --- | --- | --- | --- |
| **I** | APs + ADs versus APs + PBO (cognitive symptoms) | Treatment response  Remission  Symptoms severity  Functioning  Quality of life  Acceptability  Tolerability  Adverse events:   - endocrine (prolactin) - motor symptoms - weight gain - sedation - blood problems | Vernon et al., 2014  No data  No data  No data  No data  Vernon et al., 2014  Vernon et al., 2014  No data  Vernon et al., 2014  Vernon et al., 2014  Vernon et al., 2014  No data |  |
| **II** | APs + ADs versus APs + PBO (negative symptoms) | Treatment response  Remission  Symptoms severity  Functioning  Quality of life  Acceptability  Tolerability  Adverse events:   - endocrine (prolactin) - motor symptoms - weight gain - sedation - blood problems | Singh et al., 2010; Rummel-Kluge et al., 2010  No data  Rummel-Kluge et al., 2010  Rummel-Kluge et al., 2010  No data  Rummel-Kluge et al., 2010  Rummel-Kluge et al., 2010  No data  Rummel-Kluge et al., 2010  No data  Rummel-Kluge et al., 2010  No data |  |
| **III** | APs + ADs versus APs + PBO (depressive symptoms) | Treatment response  Remission  Symptoms severity  Functioning  Quality of life  Acceptability  Tolerability  Adverse events:   - endocrine (prolactin) - motor symptoms - weight gain - sedation - blood problems | No data  No data  Helfer et al., 2016  No data  No data  No data  Helfer et al., 2016  No data  Helfer et al., 2016  Helfer et al., 2016  No data  No data | Note: data on tolerability outcomes are from the general population of patients suffering from psychosis (indirectness) |

**GRADE tables**

**Author(s)**: Giovanni Ostuzzi

**Date**: 28/01/2016

**Question**: Antidepressant + antipsychotic compared to antipsychotic + placebo for people with psychosis-related cognitive impairment

**Setting**: humanitarian/low-resources setting

**Bibliography**: Vernon JA, Grudnikoff E, Seidman AJ, Frazier TW, Vemulapalli MS, Pareek P, Goldberg TE, Kane JM, Correll CU. Antidepressants for cognitive impairment in schizophrenia - a systematic review and meta-analysis. Schizophr Res 2014;159(2-3):385-94

| **Quality assessment** | | | | | | | **№ of patients** | | **Effect** | | **Quality** | **Importance** |
| --- | --- | --- | --- | --- | --- | --- | --- | --- | --- | --- | --- | --- |
| **№ of studies** | **Study design** | **Risk of bias** | **Inconsistency** | **Indirectness** | **Imprecision** | **Other considerations** | **antidepressant + antipsychotic** | **antipsychotic + placebo** | **Relative (95% CI)** | **Absolute (95% CI)** |  |  |
| Efficacy on cognitive impairment (follow up: mean 8.7 (SD 3.7) weeks; assessed with: composite cognition score) | | | | | | | | | | | | |
| 11 | randomised trials | serious ^1^ | not serious ^2^ | serious ^3^ | not serious ^4^ | none | 501 patients included in the analysis. Not clear how patients were allocated to each arm. The effect size significantly favours the treatment group (ADs + APs): Hedges' g 0.095, 95% CI 0.021 to 0.17, p-value=0.012. | | | | ⨁⨁◯◯ LOW | CRITICAL |
| Acceptability (follow up: mean 8.7 (SD 3.7) weeks; assessed with: n. of patients leaving the study early due to any reason) | | | | | | | | | | | | |
| 11 | randomised trials | serious ^1^ | not serious ^5^ | serious ^3^ | not serious ^6^ | none | 568 patients included in the analysis. Not clear how patients were allocated to each arm. The RR does not show relevant advantages of one treatment over another: RR 1.16, 95% CI 0.85 to 1.59, p-value=0.36. | | | | ⨁⨁◯◯ LOW | CRITICAL |
| Tolerability (follow up: mean 8.7 (SD 3.7) weeks; assessed with: n. of patients leaving the study early due to adverse events) | | | | | | | | | | | | |
| 10 | randomised trials | serious ^1^ | not serious ^5^ | serious ^3^ | not serious ^6^ | none | 540 patients included in the analysis. Not clear how patients were allocated to each arm. The RR does not show relevant advantages of one treatment over another: RR 1.79, 95% CI 0.75 to 4.27, p-value=0.19. | | | | ⨁⨁◯◯ LOW | CRITICAL |
| Motor symptoms (EPS) (follow up: mean 8.7 (SD 3.7) weeks) | | | | | | | | | | | | |
| 8 | randomised trials | serious ^1^ | not serious ^7^ | serious ^3^ | not serious ^6^ | none | 407 patients included in the analysis. Not clear how patients were allocated to each arm. The effect size does not show relevant advantages of one treatment over another: Hedges' g −0.13, 95% CI −0.33 to 0.06, p-value=0.18. | | | | ⨁⨁◯◯ LOW | IMPORTANT |
| Sedation (follow up: mean 8.7 (SD 3.7) weeks; assessed with: n. of patients experiencing sedation) | | | | | | | | | | | | |
| 4 | randomised trials | serious ^1^ | not serious ^5^ | serious ^3^ | serious ^8^ | none | 118 patients included in the analysis. Not clear how patients were allocated to each arm. The RR significantly favours the control group (APs + placebo): RR 2.91, 95% CI 1.03 to 8.17, p-value=0.04. | | | | ⨁◯◯◯ VERY LOW | IMPORTANT |
| Weight gain (follow up: mean 8.7 (SD 3.7) weeks; assessed with: n. of patients experiencing weight gain) | | | | | | | | | | | | |
| 4 | randomised trials | serious ^1^ | not serious ^9^ | serious ^3^ | not serious ^6^ | none | 300 patients included in the analysis. Not clear how patients were allocated to each arm. The RR does not show relevant advantages of one treatment over another: RR 2.08, 95% CI 0.87 to 4.97, p-value=0.10. | | | | ⨁⨁◯◯ LOW | IMPORTANT |

**CI:** Confidence interval; **MD:** Mean difference

1. The assessment blinding and the dropout rates of included studies are not reported. 1. The assessment blinding and the dropout rates of included studies are not reported. A low risk of attrition bias cannot be hypothesized since the vast majority of included studies had very small sample sizes.
2. I-squared=45%
3. None of the included trials was performed in low-resources settings.
4. The overall number of included patients is 501 and the 95% CI is relatively narrow.
5. I-squared=0%
6. The overall number of included patients is 568 and the 95% CI is relatively narrow.
7. I-squared=13%
8. The overall number of included patients is 118 and the 95% CI favours the control group.
9. I-squared=1%

**Author(s)**: Giovanni Ostuzzi

**Date**: 28/01/2016

**Question**: Antidepressant + antipsychotic compared to antipsychotic + placebo for people with psychosis-related negative symptoms

**Setting**: humanitarian/low-resources setting

**Bibliography**: Singh SP, Singh V, Kar N, Chan K. Efficacy of antidepressants in treating the negative symptoms of chronic schizophrenia: meta-analysis. Br J Psychiatry. 2010;197(3):174-9.

Rummel-Kluge C, Kissling W, Leucht S . Antidepressants for the negative symptoms of schizophrenia. Cochrane Database of Systematic Reviews 2006, Issue 3. Art. No.: CD005581.

| **Quality assessment** | | | | | | | **№ of patients** | | **Effect** | | **Quality** | **Importance** |
| --- | --- | --- | --- | --- | --- | --- | --- | --- | --- | --- | --- | --- |
| **№ of studies** | **Study design** | **Risk of bias** | **Inconsistency** | **Indirectness** | **Imprecision** | **Other considerations** | **antidepressant + antipsychotic** | **antipsychotic + placebo** | **Relative (95% CI)** | **Absolute (95% CI)** |  |  |
| Negative symptoms reduction (follow up: range 4 weeks to 12 weeks; assessed with: total score on SANS, PANSS subscale or BPRS subscale. Lower scores indicate less sever psychopathology; negative values of the SMD favour ADs + APs) | | | | | | | | | | | | |
| 22 ^14^ | randomised trials | serious ^1^ | very serious ^2^ | serious ^3^ | not serious ^4^ | none | 416 | 403 | - | MD **0.48 SD lower** (0.71 lower to 0.25 lower) | ⨁◯◯◯ VERY LOW | CRITICAL |
| Symptoms severity (short term) (follow up: range 4 weeks to 12 weeks; assessed with: endpoint BPRS score. Lower scores indicate less severe psychopathology; negative values of the MD favour ADs + APs; Scale from: 18 to 126) | | | | | | | | | | | | |
| 2 | randomised trials | not serious ^5^ | very serious ^6^ | serious ^3^ | very serious ^7^ | none | 29 | 31 | - | MD **4.89 lower** (13.27 lower to 3.5 higher) | ⨁◯◯◯ VERY LOW | IMPORTANT |
| Functioning (short term) (follow up: range 4 weeks to 12 weeks; assessed with: social withdrawal, Wing Scale Form B. Higher scores indicate better functioning; negative values of the MD favour ADs + APs) | | | | | | | | | | | | |
| 1 | randomised trials | serious ^8^ | not serious | serious ^3^ | serious ^9^ | none | 29 | 29 | - | MD **0.93 lower** (2.33 lower to 0.47 higher) | ⨁◯◯◯ VERY LOW | IMPORTANT |
| Acceptability (short term) (follow up: range 4 weeks to 12 weeks; assessed with: n. of patients leaving the study early due to any reason) | | | | | | | | | | | | |
| 3 | randomised trials | not serious ^5^ | not serious | serious ^3^ | very serious ^10^ | none | 3/45 (6.7%) | 1/45 (2.2%) | **RR 3.00** (0.35 to 26.04) | **44 more per 1000** (from 14 fewer to 556 more) | ⨁◯◯◯ VERY LOW | CRITICAL |
| Tolerability (short term) (follow up: range 4 weeks to 12 weeks; assessed with: n. of patients leaving the study early due to adverse events) | | | | | | | | | | | | |
| 2 | randomised trials | not serious ^5^ | not serious | serious ^3^ | very serious ^11^ | none | 2/32 (6.3%) | 0/32 (0.0%) | **RR 5.00** (0.26 to 97.00) | **0 fewer per 1000** (from 0 fewer to 0 fewer) | ⨁◯◯◯ VERY LOW | CRITICAL |
| Motor symptoms (short term) (follow up: range 4 weeks to 12 weeks; assessed with: n. of patients experiencing extrapyramidal events) | | | | | | | | | | | | |
| 2 | randomised trials | not serious ^5^ | not serious | serious ^3^ | very serious ^12^ | none | 2/30 (6.7%) | 0/30 (0.0%) | **RR 5.00** (0.26 to 97.00) | **0 fewer per 1000** (from 0 fewer to 0 fewer) | ⨁◯◯◯ VERY LOW | IMPORTANT |
| Sedation (short term) (follow up: range 4 weeks to 12 weeks; assessed with: n. of patients experiencing sedation) | | | | | | | | | | | | |
| 1 | randomised trials | not serious ^5^ | not serious | serious ^3^ | very serious ^13^ | none | 2/13 (15.4%) | 0/13 (0.0%) | **RR 3.00** (0.13 to 67.51) | **0 fewer per 1000** (from 0 fewer to 0 fewer) | ⨁◯◯◯ VERY LOW | IMPORTANT |

**CI:** Confidence interval; **MD:** Mean difference; **RR:** Risk ratio

1. Unclear, no data about the blinding of assessment and the dropout rates of the included studies.
2. Visual inspection of the forest plot suggests high degree of heterogeneity and this is supported by QE = 33.07, d.f.= 13, P<0.05 (MiMa function - meta-regression test).
3. None of the included trials was performed in low-resources settings.
4. The overall number of included patients is higher than 200 and the 95% CI is relatively narrow.
5. The overall dropout rate is very low for all the included studies.
6. I-squared=87%
7. The analysis included 60 patients and the 95% CI is relatively wide.
8. The dropout rate is not reported for the included study.
9. The analysis included 58 patients and the 95% CI is relatively wide.
10. The analysis includes 90 patients and the 95% CI crosses both 0.5 (appreciable benefit) and 2 (appreciable harm).
11. The analysis includes 64 patients and the 95% CI crosses both 0.5 (appreciable benefit) and 2 (appreciable harm).
12. The analysis includes 60 patients and the 95% CI crosses both 0.5 (appreciable benefit) and 2 (appreciable harm).
13. The analysis includes 26 patients and the 95% CI crosses both 0.5 (appreciable benefit) and 2 (appreciable harm).
14. Data from the meta-analysis by Helfer et al. (2016), although referred to the general population of patients suffering from schizophrenia (and not patients with predominant negative symptoms), confirm a small but statistically significant benefit in patients taking AD + AP (SMD -0.30, 95% CI -0.44 to -0.16, n=1905, N=48).

**Author(s)**: Giovanni Ostuzzi

**Date**: 14/06/2016

**Question**: Antidepressant + antipsychotic compared to antipsychotic + placebo for in patients with psychosis and pronounced depressive symptoms

**Setting**: low-resources settings

**Bibliography**: Helfer B, Samara MT, Huhn M, Klupp E, Leucht C, Zhu Y, Engel RR, Leucht S. Efficacy and Safety of Antidepressants Added to Antipsychotics for Schizophrenia: A Systematic Review and Meta-Analysis. Am J Psychiatry. 2016 Jun 10:appiajp201615081035

| **Quality assessment** | | | | | | | **№ of patients** | | **Effect** | | **Quality** | **Importance** |
| --- | --- | --- | --- | --- | --- | --- | --- | --- | --- | --- | --- | --- |
| **№ of studies** | **Study design** | **Risk of bias** | **Inconsistency** | **Indirectness** | **Imprecision** | **Other considerations** | **antidepressant + antipsychotic** | **antipsychotic + placebo** | **Relative (95% CI)** | **Absolute (95% CI)** |  |  |
| Symptoms severity (follow up: median 8 weeks; assessed with: various rating scales score; negative SMDs favour AD + AP) | | | | | | | | | | | | |
| 14 | randomised trials | not serious ^1^ | serious ^2^ | serious ^3^ | not serious ^4^ | none | 417 | 360 | - | SMD **0.34 SD lower** (0.58 lower to 0.09 lower) | ⨁⨁◯◯ LOW | IMPORTANT |
| Tolerability (follow up: median 8 weeks; assessed with: Dropouts due to adverse events) | | | | | | | | | | | | |
| 31 | randomised trials | not serious ^5^ | not serious ^6^ | very serious ^7^ | not serious ^8^ | none | 51/766 (6.7%) | 30/727 (4.1%) | **RR 1.36** (0.88 to 2.10) | **15 more per 1.000** (from 5 fewer to 45 more) | ⨁⨁◯◯ LOW | CRITICAL |
| Weight gain (follow up: median 8 weeks; assessed with: n. of patients with relevant weight gain at study endpoint) | | | | | | | | | | | | |
| 8 | randomised trials | not serious ^5^ | not serious ^9^ | very serious ^7^ | not serious ^10^ | none | 52/240 (21.7%) | 61/227 (26.9%) | **RR 0.86** (0.64 to 1.16) | **38 fewer per 1.000** (from 43 more to 97 fewer) | ⨁⨁◯◯ LOW | IMPORTANT |
| Motor symptoms (follow up: median 8 weeks; assessed with: n. of patients with parkinsonism at study endpoint) | | | | | | | | | | | | |
| 7 | randomised trials | not serious ^5^ | not serious ^6^ | very serious ^7^ | not serious ^11^ | none | 40/158 (25.3%) | 37/158 (23.4%) | **RR 1.14** (0.79 to 1.63) | **33 more per 1.000** (from 49 fewer to 148 more) | ⨁⨁◯◯ LOW | IMPORTANT |

**CI:** Confidence interval; **SMD:** Standardised mean difference; **RR:** Risk ratio

1. The attrition bias was evaluated to be low for 8 out of 14 studies, uncertain for 2 and high for 4 studies. The risk of performance and detection bias was evaluated to be low for the vast majority of studies.
2. I-squared=59%
3. None of the included trials was performed in low-resources settings.
4. The overall number of included patients is 777 and the 95% CI is narrow.
5. Attrition, detection and performance bias were evaluated to be low for the vast majority of studies.
6. I-squared=0%
7. None of the included trials was performed in low-resources settings. Further, data on tolerability outcomes are from the general population of patients suffering from psychosis.
8. The overall number of included patients is 1493 and the 95% CI is narrow.
9. I-squared=24%
10. The overall number of included patients is 467 and the 95% CI is relatively narrow.
11. The overall number of included patients is 316 and the 95% CI is relatively narrow.
